# Restrictions of VC and DLCO in relation to asbestos-related computed tomographic findings quantified by ICOERD-based parameters

**DOI:** 10.1186/s12890-022-02022-x

**Published:** 2022-06-20

**Authors:** Lennart Ströker, Kersten Peldschus, Robert Herold, Volker Harth, Alexandra Marita Preisser

**Affiliations:** 1 Institute for Occupational and Maritime Medicine (ZfAM), University Medical Center Hamburg-Eppendorf (UKE), Hamburg, Germany; 2 Department of Diagnostic and Interventional Radiology and Nuclear Medicine, University Medical Center Hamburg-Eppendorf (UKE), Hamburg, Germany

**Keywords:** Asbestosis, Lung function, Vital capacity, CO diffusion capacity, ICOERD, Pleural plaques, Fibrosis, Parenchymal bands, Subpleural curvilinear lines, Rounded atelectasis

## Abstract

**Background:**

Even almost 30 years after the ban on the use of asbestos in Germany, the effects of asbestos are still highly relevant in everyday clinical practice in occupational medicine. The aim of this study was to further investigate the significance of essential parameters of both pulmonary function diagnostics and imaging techniques (low-dose HR-TCT) for the prevention and early detection of asbestos-related morphological and functional lung changes.

**Methods:**

Data from spirometry, body plethysmography and diffusion capacity, as well as CT images of the thorax, were retrospectively studied from 72 patients examined between 2017 and 2019 at the Institute for Occupational and Maritime Medicine (ZfAM), Hamburg, Germany. The subjects were divided into four subgroups according to the presence of comorbidities (concomitant cardiac diseases, obstructive ventilatory disorder, pulmonary function pattern consistent with emphysema, and no other pulmonary or cardiac diseases). These subgroups were analysed in addition to the overall collective. The CT images were evaluated according to the International Classification of Occupational and Environmental Respiratory Diseases (ICOERD) with radiological expertise. In addition, some asbestos-related parameters were newly quantified, and corresponding scores were defined based on ICOERD. Statistical analysis included the use of correlations and fourfold tables with calculation of Spearman's rho (ρ), Cohen’s κ, and accuracy.

**Results:**

Vital capacity (VC) is slightly reduced in the total collective compared to the normal population (mean 92% of predicted value), while diffusion capacity for CO (D_LCO_) shows predominantly pathological values, mean 70% of the respective predicted value. The CO transfer coefficient (D_LCO_/VA), which refers to alveolar volume (VA), also shows slightly decreased values (mean 87% pred.). Seventy-nine percent of patients (n = 57) had signs of pulmonary fibrosis on CT scans, and pleural plaques appeared in 58 of 72 patients (81%). Of the newly quantified additional parameters, particularly frequently described findings are subpleural curvilinear lines (SC, n = 39) and parenchymal bands (PB, n = 29). VC correlates well with the expression of pleural plaques (ρ = − 0.273, *P* < 0.05), and D_LCO_ measures show a better correlation with fibrosis score (ρ = − 0.315, *P* < 0.01). A third, newly developed score, which includes the extent of pleural plaques and additional subpleural parameters instead of fibrosis parameters, shows significant correlations for both VC and D_LCO_ (ρ = − 0.283, − 0.274, resp.; both *P* < 0.05).

**Discussion:**

The importance of spirometry (VC) and diffusion capacity measurement (D_LCO_) as essential diagnostic procedures for the early detection of asbestos-related changes ‒ also including patients with relevant concomitant cardiac or pulmonary diseases ‒ was confirmed. Significant and better correlations between lung function changes (VC and D_LCO_) and abnormal CT findings are seen when parenchymal bands (PB), subpleural curvilinear lines (SC), and rounded atelectasis (RA) are quantitatively included into the evaluation, in addition to assessing the extent of pleural plaques alone. Therefore, when assessing CT images according to ICOERD, these parameters should also be quantified.

**Supplementary Information:**

The online version contains supplementary material available at 10.1186/s12890-022-02022-x.

## Background

Due to the extensive application of asbestos fibres in recent decades until the ban of use in Germany in 1993 and in the EU in 2005 and based on the high latency period between exposure and symptom onset of up to 40 years, asbestos-related diseases still rank very high among work-related diseases. In Germany, a total of 10,038 cases of asbestos-related occupational diseases were confirmed from 2017 to 2019 [1]. This represents 9% of all confirmed occupational diseases in this period, while asbestos-related deaths (4896) account for 65% of total deaths due to occupational diseases during this time, with an upward trend [1]. Due to the long latency period and the widespread industrial as well as private application, a high number of unreported cases must be assumed.

Typical consequences of years of asbestos exposure are, in addition to the malignant diseases bronchial carcinoma or pleural mesothelioma, in particular the development of pulmonary fibrosis, pleurisy or the manifestation of pleural plaques [2]. These changes result in restrictive lung dysfunction with a reduction of all volumes with normal to little change in the FEV1/FVC ratio. There is also a reduction in the diffusion capacity for CO (D_LCO_) [3]. In diagnostic imaging, low-dose high-resolution computed tomography of the chest (HR-TCT) is more suitable than chest X-ray examinations for assessing typical pathological processes of the lung parenchyma and pleura [4, 5]. Since 2017, low-dose HR-TCT has been established in Germany as an important diagnostic tool for the early detection of asbestos-related diseases in formerly asbestos-exposed individuals [6].

Radiologic findings indicative of pulmonary fibrosis include irregular, linear and reticular opacities seen as thickened interlobular septa or as intralobular nonseptal lines. Honeycombing structures are definite signs of pulmonary fibrosis. Pleural-parietal plaques are considered specific for previous asbestos exposure; they usually occur bilaterally and multilocularly spread over the pleura [7, 8]. Other highly asbestos-related findings of the lung and pleura include: subpleural curvilinear lines (SC), parenchymal bands (PB), rounded atelectases (RA), and effusion (EF). They are indicative of early-stage pulmonary fibrosis but are also rather difficult to diagnose [7–9].

The typical changes caused by asbestos exposure ‒ fibrosis and pleural plaques ‒ can result in a lower vital capacity (VC) in pulmonary function tests. VC and one-second forced expiratory volume (FEV1) are established parameters in non-invasive diagnostics. The measurement of diffusion capacity (D_LCO_) is also suitable for the early recognition of changes detectable in HR-TCT after exposure to asbestos. D_LCO_ is a sensitive parameter for detecting asbestos-related fibrosis, whereas the transfer coefficient D_LCO_/VA (Krogh factor, KCO) is less informative [10, 11]. In addition to pulmonary fibrosis and pleural plaques, other radiologic changes of the subpleural lung parenchyma, such as SC, PB, or RA, may also be associated with pulmonary function restrictions [10].

The aim of the study presented here is to confirm and quantify more precisely the relationship between findings in HR-TCT and asbestos-typical changes in lung function to improve secondary prevention of asbestos-related diseases. Furthermore, it is shown that the non-invasive and economically favourable method of pulmonary function testing plays a relevant role in the early detection of asbestos-associated pathological processes.

## Methods

For this study, data from a patient collective of 77 formerly asbestos-exposed patients examined from 2017 to 2019 at the Central Institute for Occupational and Maritime Medicine (ZfAM), Hamburg (Germany), were retrospectively analysed. An occupational and general medical history (incl. number of pack years (py)), physical examination results (incl. age, height, weight, BMI), as well as a current HR-TCT examination were collected. Pulmonary function tests and TCT were performed as described below. Partially, blood tests (with haemoglobin value (Hb) and percentage CO-Hb) were available. In the case of bronchial obstruction, bronchodilation was performed.

Based on the data collected, patients were divided into four subgroups.

- Subgroup 1: Patients with relevant concomitant cardiac diseases.
- Subgroup 2: Patients with obstructive ventilatory disorder (after bronchodilation).
- Subgroup 3: Patients with pulmonary function changes or radiological findings in terms of emphysema.
- Subgroup 4: Patients without other relevant pulmonary or cardiac diseases.

Five of the 77 patients were excluded from the study because of incomplete records or missing CT data; this included the only woman in the study group. Thus, an all-male patient collective of 72 persons was evaluated.

The assignment to the subgroup 1 concomitant cardiac diseases (sub-1) was mainly based on the medical history of the subjects and the results of the physical examination (performed according to the guidelines of the German Society of Cardiology (DKG)) [12–14]. Cardiac conditions in our study collective were primarily chronic heart failure, coronary heart disease, and chronic atrial fibrillation. For assignment to the subgroup with fixed obstructive ventilatory disorder (sub-2), at least one of the following criteria had to be met: (1) decrease in FEV1/FVC ratio < lower limit of normal (LLN, corresponding to the Global Lung Initiative (GLI) 5th percentile) [15, 16], or (2) increase in specific airway resistance sRt > 1.2 kPa s [17].

Criteria for classification into the subgroup 3 lung function pattern or HR-TCT findings appropriate for pulmonary emphysema (sub-3) were (1) increase in residual volume (RV) and fraction of residual volume to total lung capacity (RV/TLC) > upper limit of normal (ULN) after bronchodilator test or (2) radiologically visible signs of emphysema [15]. The subgroup 4 with no other pulmonary or cardiac diseases (sub-4) includes the patients who could not be assigned to any of the groups mentioned above.

### Pulmonary function test

Spirometry and body plethysmography were performed according to the European Respiratory Society (ERS), American Thoracic Society (ATS), and the German guideline for standardization of spirometry [18–21]. Proper evaluation of lung function requires at least three artefact-free breathing manoeuvres with acceptable reproducibility in which the best two attempts for FEV1 and FVC do not differ by more than 5% [21]. The highest result of the performed breathing manoeuvres was used for data analysis. The interpretation of the results was based on the GLI reference values for spirometry and the European Coal and Steel Community (ECSC) reference values for body plethysmography [22, 23]. The following lung function parameters were analysed: maximum vital capacity (VC_max_), forced expiratory vital capacity (FVC), one-second forced expiratory volume (FEV1), specific respiratory resistance (sRt), functional residual capacity (FRCpleth.), total lung capacity (TLC), residual volume (RV) and RV/TLC ratio as well as diffusion capacity of the lung for carbon monoxide (D_LCO_) and transfer coefficient D_LCO_/VA (KCO, Krogh-Factor). Measurements of D_LCO_ were carried out using the single-breath method (D_LCO_-SB) with the reference values according to Cotes et al. [24] as recommended by Graham et al. [25].

Haemoglobin (Hb) values were determined in 33 patients; here, D_LCO_ was corrected (according to MacIntyre et al.): D_LCO_ (corrected D_LCO_) = D_LCO_ × (10.22 + Hb/1.7 × Hb) [26]. In the other subjects, D_LCO_ was set with a fixed Hb value of 14.6 g/dl.

### Computed tomography examination

The acquisition of HR-TCT examinations was carried out in compliance with a defined technical standard with uniform acquisition and reconstruction parameters. During the evaluation of the CT data sets, the slice thickness, the number of slices, the type of reconstruction, the dose, and the positioning of the patient were documented in accordance with the recommendations of the Working Group for Diagnostic Radiology in Occupational and Environmental Diseases (AG DRauE) of the German Roentgen Society [27]. Based on these technical criteria, each dataset was rated with a quality criterion from 1 to 4 [28].

The CT datasets were semi-quantitatively assessed using the International Classification of Occupational and Environmental Respiratory Diseases (ICOERD) according to Hering et al. [8]. ICOERD is considered the current standard for CT assessment of pneumoconiosis [29]. It is used to evaluate both the lungs and pleura. Lung changes are assessed by round opacities, irregular and/or linear opacities (divided into intra- and interlobular), ground glass, honeycombing, consolidations, and emphysema formations.

The ICOERD can be used to assign a score for each of the categories mentioned above: in the data sheet, the lung is divided into an upper (U), middle (M) and lower (L) field per side. Up to three points can be awarded per field and side depending on the disease-related parenchymal changes. Thus, values from 0–18 points are possible in the evaluation of lung changes for each category. For a reliable diagnosis of asbestosis, bilateral irregular and/or linear opacities with a score ≥ 2 or bilateral honeycombing with a score ≥ 2 are required findings in HR-TCT (according to Helsinki Criteria) [30].

With regard to the pleura, visceral findings are distinguished from parietal findings. The parietal type can show flat, spindle- or tableland-shaped thickenings and is typical of asbestos-related changes. Visceral findings occur as circumscribed thickening of the pleura and may also have other causes but do not rule out an asbestos-related genesis [7, 8]. Pleural findings are classified in the ICOERD according to the location of the pleural plaques in the right and left side as well as in the upper (U), middle (M), and lower (L) field, resulting in a possible pleural score of 0–6 points.

The relevant scores for the presence of asbestos-typical findings, as also confirmed in previous studies, are the scores for irregular/linear opacities (fibrosis score, Score A) and pleural plaques (pleural plaques score, Score B) [10]. In addition to the categories described, it is possible for the evaluator to mark additional pathological findings on the ICOERD sheet. Major pleuropulmonary changes associated with asbestos include:

- Parenchymal bands (PB): Orthogonally oriented fibrosis strands directly adjacent to the pleura.
- Subpleural curvilinear lines (SC): Subpleural linear densifications running parallel to the pleura.
- Rounded atelectasis (RA): Atelectasis with scarring retractions of the visceral pleura and curling of adjacent lung tissue.
- Effusion, free or loculated (EF): Pleural effusion [31].

Each of these additional parameters was assigned a possible score from 0–18 for this study evaluation; the original sheet provides only dichotomous quantification for this purpose. The assessment of the point values (0–18) for these parameters was performed in analogy to the evaluation of parenchymal lung changes described above. Subsequently, another score was developed using the newly quantified parameters (Score C): pleural plaques + (PB + SC + RA). Thus, values from 0–60 points are possible in this score. Other ICOERD-based scores were tested, including addition and/or multiplication of the different individual parameters. Since this did not reveal any further meaningful results, these approaches were not pursued.

All HR-TCTs were evaluated by the same radiologist. In 50 patients, pulmonary function testing and CT imaging were performed within 30 days, 11 subjects received examinations within a period of 30 to 60 days, and in another 11 subjects, the interval between examinations was more than 60 days (mean 44 days, SD 67, median 18 days).

### Statistical analysis

Descriptive statistics of demographic parameters, pulmonary function values, and ICOERD findings were recorded for the entire cohort and for each of the four subgroups. For the total collective, tests for normal distribution were performed for the above parameters using the Shapiro–Wilk test. Lung function parameters VC_max_, D_LCO_ and D_LCO_/VA were tested for statistical correlation to Scores A, B, and C from CT findings. To account for and compensate for individual differences in body size and age, pulmonary function parameters were presented as a percentage of the respective reference value [24]. Correlations between lung function values and CT findings were applied using Spearman’s rho correlation coefficient (ρ) and were tested for significance. Further analyses were performed using fourfold tables. For this purpose, subjects were classified as healthy or diseased according to radiological signs of pulmonary fibrosis or pleural plaques in CT. Findings with an ICOERD Score A ≥ 2 were considered fibrosis, and the presence of pleural plaques was considered confirmed with a Score B ≥ 1 [10]. Pulmonary function values were assessed according to exceeding or falling below the LLN (lower limit of normal). Accuracy and Cohen’s kappa (κ) were calculated accordingly. The IBM® SPSS® Statistics Version 26 program was used for the statistical analysis.

### Ethics approval and informed consent

All participants gave written (informed) consent to clinical studies. According to the Ethics Committee of the Hamburg Medical Association, an ethics vote was not required due to the in-house research and the retrospective evaluation and analysis of the anonymized data. The authors confirm that all methods were carried out in accordance with relevant guidelines and regulations.

## Results

### Characteristics of the study population

The examination findings of 72 men were evaluated; the study population did not include women. The individuals were assigned to the pre-established subgroups: 41 subjects with concomitant cardiac diseases (sub-1), 24 with obstructive ventilatory disorder (sub-2), 25 with lung function pattern or HR-TCT findings appropriate for pulmonary emphysema (sub -3), and 14 subjects without other pulmonary or cardiac diseases (sub-4). As a result of multimorbidity, 25 individuals were assigned to multiple subgroups. Subgroups 2 and 3 in particular showed a wide overlap due to the similarities in genesis (17 subjects with both obstructive ventilatory disorder and pulmonary emphysema). However, some patients with radiological signs of emphysema did not show pulmonary obstruction in pulmonary function testing (n = 9). The subgroups were assessed and compared in addition to the overall collective.

The total collective is normally distributed with regard to the variables age, weight, height, BMI value, and haemoglobin value. The number of pack-years ranges from 0 to 71 (median 34 years). The dose of asbestos exposure was not recorded because previous studies have not found a relationship between exposure duration and lung function values or only rough dose–response relationships [10, 32]. The evaluation of occupational histories reveals that patients were exposed to asbestos in 22 different occupations. The most common occupations identified were locksmith (n = 12), automotive mechanic (n = 10), and electrician (n = 8) (Additional file 1). Table 1 shows the main demographic data of the study population.

**Table 1:** Demographic data of the patient collective (n = 72)

Variable	n	Mean	SD
Age [years]	72	69.9	8.2
Weight [kg]	72	89.5	16.3
Height [cm]	72	175.3	7.5
BMI [kg/m^2^]	72	28.9	5.1
Cigarette smoking [py]^a^	69	32.2	18.4
Hb [g/dl]	33	15.2	1.8

*py* packyears, *Hb* haemoglobin, *SD* standard deviation

^a^Sixty-three subjects were smokers or former smokers

### Pulmonary function results

The most important lung parameters from spirometry, body plethysmography and CO diffusion measurement are listed in Table 2. For VC, TLC, FVC, D_LCO_ and D_LCO_/VA, the overall collective shows a normal distribution.

**Table 2:** Results of pulmonary function testing and diffusion measurement

Variable	Total (n = 72)		sub-1 (n = 41)		sub-2 (n = 24)		sub-3 (n = 25)		sub-4 (n = 14)	
	Mean	SD	Mean	SD	Mean	SD	Mean	SD	Mean	SD
VC_max_ [L]	3.78	0.93	3.53	0.86	3.35	0.81	3.59	0.95	4.43	0.79
VC_max_ [%pred.]	91.97	17.30	87.37	16.40	84.25	17.13	90.68	19.77	101.64	13.48
sRt [kPa s]	1.35	1.06	1.39	1.26	2.26	1.39	1.96	1.49	0.97	0.37
sRt [%pred.]	117.85	102.53	124.10	125.65	201.67	141.30	171.20	145.65	82.36	31.81
TLC [L]	7.27	1.51	6.91	1.44	7.76	1.69	7.80	1.69	7.36	1.17
TLC [%pred.]	104.76	20.19	99.85	18.60	114.58	25.65	115.16	25.12	103.14	11.89
FRC_pleth_ [L]	4.10	1.18	3.84	0.95	4.76	1.49	4.83	1.46	3.66	0.83
FRC_pleth_ [%pred.]	116.54	48.17	112.44	55.44	131.33	40.91	133.48	39.19	99.79	19.90
RV [L]	3.45	1.26	3.32	1.27	4.41	1.50	4.22	1.59	2.93	0.61
RV [%pred.]	132.65	46.25	125.66	46.32	169.25	52.96	161.52	57.27	115.43	21.81
RV/TLC [%]	47.04	10.39	47.61	11.18	56.08	9.03	53.14	11.00	39.83	4.96
RV/TLC [%pred.]	114.17	23.00	113.32	24.86	134.79	16.34	127.44	23.43	101.00	11.63
FVC [L]	3.69	0.96	3.44	0.89	3.20	0.88	3.46	1.01	4.38	0.80
FVC [%pred.]	88.78	17.35	84.80	16.76	79.29	17.14	86.28	19.94	99.29	13.14
FEV1 [L]	2.65	0.82	2.51	0.79	1.98	0.66	2.23	0.83	3.39	0.54
FEV1 [%pred.]	83.69	21.78	81.83	22.23	64.25	18.47	73.24	24.70	99.00	13.13
D_LCO_^a^ [mmol min^−1^ kPa^−1^]	6.19	1.83	5.74	1.61	5.28	1.32	5.14	1.70	8.04	1.44
D_LCO_^a^ [%pred.]	69.50	16.40	65.91	16.10	60.97	12.31	59.49	14.97	86.06	12.14
D_LCO_/VA^a^ [mmol min^−1^ kPa^−1^]	1.11	0.23	1.08	0.23	0.99	0.17	0.93	0.20	1.34	0.15
D_LCO_/VA^a^ [%pred.]	86.91	17.37	85.98	18.76	78.49	14.65	73.93	15.60	101.40	7.43

Some patients were assigned to several subgroups

sub-1: Pat. with concomitant cardiac diseases; sub-2: Pat. with obstructive ventilatory disorder; sub-3: Pat. with lung function pattern indicating pulmonary emphysema; sub-4: Pat. without other pulmonary or cardiac diseases

*VC*_max_ maximum vital capacity, *sRt* total specific respiratory resistance, *TLC* total lung capacity, *FRC*_pleth_ functional residual capacity in body plethysmography, *RV* residual volume, *RV/TLC* share of residual volume in TLC, *FVC* forced expiratory vital capacity, *FEV1* one-second forced expiratory volume, *D*_LCO_ diffusion capacity for CO, *D*_LCO_/VA D_LCO_ in relation to alveolar volume, %*pred*. % of the respective predicted value, *SD* standard deviation

^a^for D_LCO_ and D_LCO_/VA n = 69 (including sub-1 n = 39, sub-2 n = 23, sub-3 n = 24, sub-4 n = 13)

**Fig. 1:**
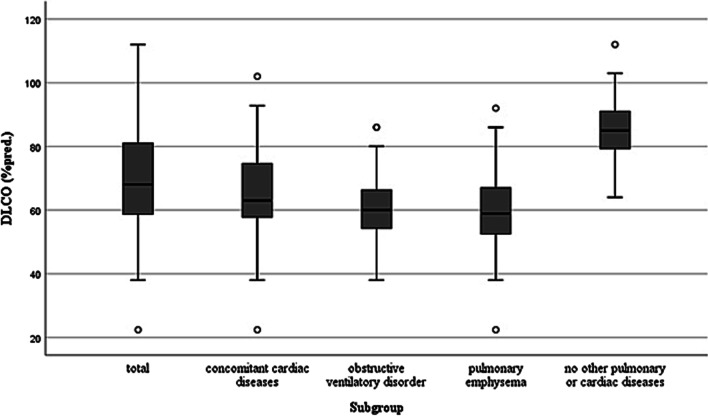
Box plot presentation of D_LCO_ values sorted by subgroups

In the total collective, there is a mean VC of 3.78 L. On average, the VC shows a slight undercut of the respective predicted value (mean 92% pred.), while the results of the CLT are in line with the norm (mean 105% pred.). On average, sRt is elevated (mean 1.35 kPa s). In addition to a reduced FEV1 (mean 84% pred.), an increased residual volume is also notable (mean 133% pred.). Subjects in the subgroups obstructive ventilatory disorder (sub-2) and lung function or HR-TCT findings indicating emphysema (sub-3) have correspondingly elevated RV/TLC values (mean 135% pred. and 127% pred., resp.). In subjects without further pulmonary or cardiac diseases, there are no relevant deviations from the respective predicted values, whereas the results of patients with cardiac diseases correspond to the average values of the total collective (see Table 2). CO diffusion capacity (D_LCO_) shows decreased values in the total study population (mean 70% pred.), and patients without further pulmonary or cardiac diseases also have lower results compared to the respective predicted values (mean 86% pred.). Individuals from the subgroup lung function or HR-TCT findings indicating emphysema (sub-3) have the worst diffusion capacity (mean 59% pred.). Figure 1 presents the box plots of the measured D_LCO_ values for the subgroups.

Evaluation of the transfer coefficient D_LCO_/VA (KCO) in the whole collective shows better results (mean 87% pred.) Again, patients with pulmonary emphysema have the worst values, while subjects without other pulmonary or cardiac diseases are in the reference range on average (see Table 2).

### Radiology results

Of the 72 HR-TCT data sets, 59 could be assigned to quality 1 (no assessment limitation), 8 to quality 2 (slightly limited assessment), 3 to quality 3 (significantly limited assessment), and 0 to quality 4 (no assessment possible). Quality information was not available for two patients.

Evaluation of the CT data sets according to ICOERD reveals the following results: 57 of the 72 patients show radiologic findings indicative of pulmonary fibrosis (i.e., ≥ 2 points in fibrosis score = Score A). The extent of radiologically detectable irregular/linear opacities is rather low (median 3 points, range 0–10 points), and the maximum score in the patient collective is 10 points (n = 1). Approximately 65% of all patients have score values between 2 and 4 points (out of a maximum possible 18 points). Honeycombing as the final form of pulmonary fibrosis is seen in only two cases, each scored as 1 point.

Pleural plaques (Score B) were found in 58 patients (median 4 points, range 0–6 points); the most frequently assigned and highest score is 6 points (n = 17), followed by 5 and 4 points (each n = 10). Fourteen of these 17 patients with a score of 6 (82.4%) have concomitant cardiac diseases. Subjects with obstructive ventilatory disorder (sub-2) or a lung function pattern indicating emphysema (sub-3) show a homogeneous distribution of score values (both median 3 points, range 0–6 points). In patients without other pulmonary or cardiac diseases, four subjects without detectable pleural plaques were identified (median 2 points, range 0–6 points).

Of the whole study collective, nine subjects show signs of fibrosis but do not have pleural plaques. Ten patients have pleural plaques without fibrosis. Both signs of fibrosis and pleural plaques were found in 53 patients.

The frequency distribution of the newly observed additional parameters is shown in Table 3. Subpleural curvilinear lines (n = 39) and parenchymal bands (n = 29) show the highest prevalence with the widest range in the results, whereas the occurrence of RA (n = 9), and EF (n = 5) is rarely noted. The maximum values of SC and PB are 5 points each. Of 72 patients, 22 have no findings of an additional parameter.

**Table 3:** Results of the ICOERD evaluation (n = 72)

ICOERD value	Frequency of value (% of n = 72)					
	Fibrosis (Score A)	PP (Score B)	PB	SC	RA	EF
0	2 (2.8)	14 (19.4)	43 (59.7)	33 (45.8)	63 (86.1)	67 (93.1)
1	13 (18.1)	4 (5.6)	10 (13.9)	18 (25.0)	7 (9.7)	
2	18 (25.0)	10 (13.9)	9 (12.5)	8 (11.1)	1 (1.4)	3 (4.2)
3	12 (16.7)	7 (9.7)	4 (5.6)	7 (9.7)	1 (1.4)	2 (2.8)
4	17 (23.6)	10 (13.9)	3 (4.2)	4 (5.6)		
5	4 (5.6)	10 (13.9)	3 (4.2)	2 (2.8)		
6	3 (4.2)	17 (23.6)				
7–10	3 (4.2)					
Median	3	4	0	1	0	0
Range	0–10	0–6	0–5	0–5	0–3	0–3

*PP* pleural plaques, *PB* parenchymal bands, *SC* subpleural curvilinear lines, *RA* rounded atelectasis, *EF* effusion

### Correlation of pulmonary function parameters with fibrosis score (Score A) and pleural plaque score (Score B)

The three parameters VC, D_LCO_ and D_LCO_/VA were correlated with the abovementioned ICOERD-based Scores A and B as well as the newly developed Score C.

#### Pulmonary fibrosis

The 57 patients with signs of fibrosis on CT show overall lower values for VC, D_LCO_, and D_LCO_/VA than patients without corresponding radiological findings (Table 4). For vital capacity, the Spearman correlation with fibrosis expression is ρ = − 0.168 (n.s.). This relationship is particularly prominent in patients with obstructive ventilatory disorder (sub-2), with a correlation of ρ = − 0.551 (*P* < 0.01). In all other subgroups, there are slight, nonsignificant correlations of pulmonary fibrosis and VC at best (Table 4.). Of 72 patients from the overall cohort, only 16 in total have a VC below LLN (Additional file 2); of these, 14 show fibrosis on computed tomography (positive predictive value (PPV) 87.5%). Forty-three subjects have radiological signs of fibrosis and regular VC.

**Table 4:** Relation between VC, D_LCO_, and D_LCO_/VA and the degree of pulmonary fibrosis according to the ICOERD classification

Group	Fibrosis	VC [%pred.]			D_LCO_ [%pred.]			D_LCO_/VA [%pred.]		
		n	Mean	SD	n	Mean	SD	n	Mean	SD
Total	None	15	97.00	13.25	14	74.97	16.48	14	90.16	18.99
	Yes^a^	57	90.65	18.08	55	68.11	16.24	55	86.09	17.02
	Correlation ρ^b^		− 0.168			− 0.315*			− 0.172	
sub-1	None	6	87.17	12.24	6	70.05	20.76	6	91.52	23.50
	Yes^a^	35	87.40	17.16	33	65.16	15.38	33	84.97	18.03
	Correlation ρ^b^		− 0.098			− 0.297			− 0.176	
sub-2	None	5	102.80	16.15	5	69.00	11.49	5	75.80	7.12
	Yes^a^	19	79.37	14.00	18	58.74	11.88	18	79.24	16.22
	Correlation ρ^b^		− 0.551*			− 0.297			0.150	
sub-3	None	5	107.40	11.78	5	74.80	14.60	5	79.00	11.29
	Yes^a^	20	86.50	19.31	19	55.46	12.53	19	72.59	16.54
	Correlation ρ^b^		− 0.251			− 0.230			0.131	
sub-4	None	4	98.75	2.63	3	85.10	5.85	3	106.03	3.56
	Yes^a^	10	102.80	15.96	10	86.35	13.73	10	100.01	7.84
	Correlation ρ^b^		0.284			0.099			− 0.507	

Some patients were assigned to several subgroups

sub-1: Pat. with concomitant cardiac diseases; sub-2: Pat. with obstructive ventilatory disorder; sub-3: Pat. with lung function pattern indicating pulmonary emphysema; sub-4: Pat. without other pulmonary or cardiac diseases

*VC* vital capacity, *D*_LCO_ diffusion capacity for CO, *D*_LCO_/*VA* D_LCO_ in relation to alveolar volume, %*pred*. % of the respective predicted value, *SD* standard deviation

^*^Significant (*P* < 0.01)

^a^Indicates fibrosis score (Score A) ≥ 2

^b^Correlation ρ of pulmonary fibrosis (Score A) with lung function parameters

**Fig. 2:**
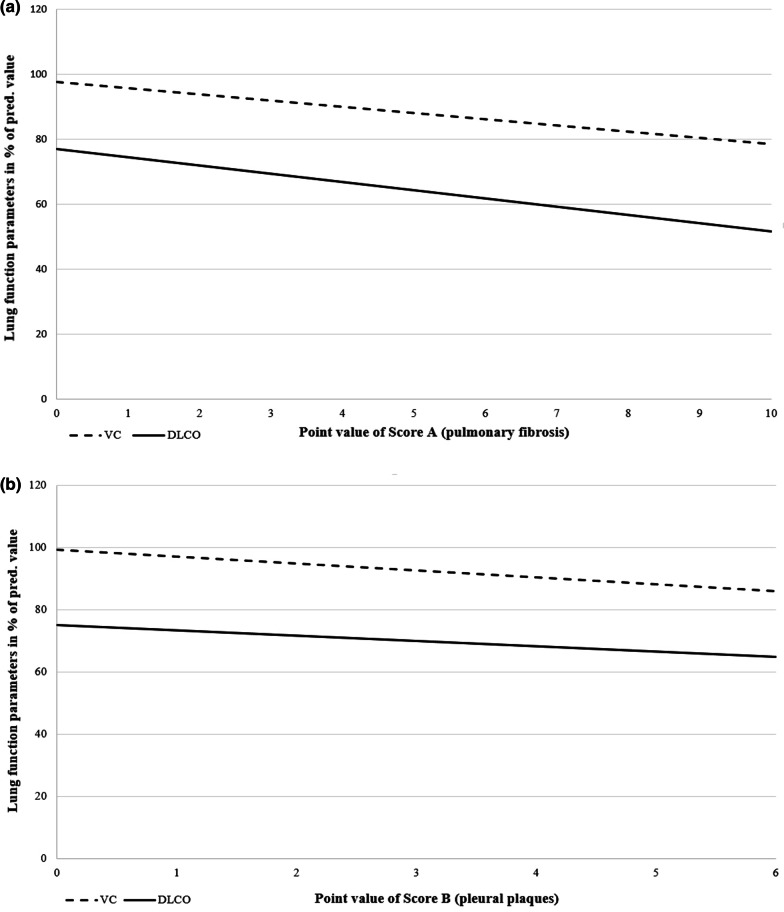
**a** Comparison of vital capacity and diffusion capacity with pulmonary fibrosis (point value of Score A). VC: n = 72, ρ = -0.168, n.s.; D_LCO_: n = 69, ρ = -0.315, *P* < 0.01. **b** Comparison of vital capacity and diffusion capacity with pleural plaques (point value of Score B). VC: n = 72. ρ = -0.273, *P* < 0.05; D_LCO_: n = 69. ρ = -0.175, n.s

Diffusion capacity D_LCO_ proves to be the most indicative measurement parameter with a correlation to fibrosis of ρ = − 0.315 (*P* < 0.01) (Fig. 2a) and a nearly seven percent difference in D_LCO_ between patients with and without fibrosis (mean 68.1% pred. and mean 75% pred., resp.). In subgroup analysis, especially in subjects with concomitant cardiac diseases (sub-1) and in subjects with obstructive ventilatory disorder (sub-2), meaningful correlations stand out (each ρ = − 0.297, n.s.). Evaluation of the fourfold tables shows that diffusion measurement detects fibrotic remodeling processes with a sensitivity of 73%. Fifteen subjects with fibrosis have a VC ≥ LLN. Forty of 45 patients with D_LCO_ < LLN have pulmonary fibrosis (PPV 89%). With a resulting 71% accuracy, D_LCO_ has good informative value for the diagnosis of fibrosis. According to Landis and Koch, the relationship between these parameters is "fair" with a Cohen’s κ of 0.29 [33]. High agreement with D_LCO_ is also found in the fourfold tables in patients with obstructive ventilatory disorder and in patients with lung function pattern indicating emphysema (accuracy 78%, 88%, resp.; κ = 0.31, 0.59, resp.).

Evaluation of D_LCO_/VA shows only a weak correlation with fibrosis score (Score A) for the whole study group (ρ = − 0.172, n.s.). Only in patients without other pulmonary or cardiac diseases (sub-4), D_LCO_/VA decreases with increasing fibrosis, with a moderate correlation (ρ = − 0.507; n.s.). In all other subgroups, the correlation values between fibrosis and D_LCO_/VA are inhomogeneous. Accuracy and Cohen’s κ show no explanatory power of D_LCO_/VA, neither for the overall collective (28%, κ = − 0.01, resp.) nor for the subgroups.

In subjects with signs of fibrosis but without pleural plaques, the correlations of fibrosis with VC, D_LCO_ and D_LCO_/VA do not provide significant results (ρ = 0.365, − 0.183, 0.183, resp.; all n.s.).

#### Pleural plaques

Eighty-one percent (58 of 72) of all patients have pleural plaques and show a slightly lower vital capacity than patients without corresponding radiological findings (mean 90.7% pred. and 97.4% pred., resp.). The extent of pleural plaques shows a significant correlation with VC (ρ = − 0.273, *P* < 0.05) (Fig. 2b). In line with the outcomes of pulmonary fibrosis analysis, patients with obstructive ventilatory disorder (sub-2) show the strongest relationship with the decrease in VC (ρ = − 0.666, *P* < 0.01); for this group, a good accuracy of 63% is also found with a fair "Strength of Agreement" (κ = 0.33). Patients with pleural plaques but no signs of fibrosis also show a meaningful correlation between VC and the extent of pleural plaques (ρ = − 0.403, n.s.). The PPV for the presence of pleural plaques in reduced VC is 94%. Forty-three subjects with pleural plaques show a normal VC.

In the overall cohort, D_LCO_ shows only a weak correlation with pleural plaque expression (ρ = − 0.175, n.s.) (Table 5). In the patient group with no other pulmonary or cardiac diseases (sub-4), the negative association becomes more evident (ρ = − 0.25, n.s.). In the fourfold table of the total study group, however, D_LCO_ shows a higher meaning for the presence of pleural plaques with 61% accuracy than VC (39% accuracy). The PPV for the presence of pleural plaques in reduced CO diffusion capacity is 82%. Nineteen patients with pleural plaques have a D_LCO_ ≥ LLN.

**Table 5:** Relation between VC, D_LCO_, and D_LCO_/VA and the degree of pleural plaques according to the ICOERD classification

Group	Pleural plaques	VC [%pred.]			D_LCO_ [%pred.]			D_LCO_/VA [%pred.]		
		n	Mean	SD	n	Mean	SD	n	Mean	SD
Total	None	14	97.36	13.20	13	71.85	17.18	13	84.23	19.00
	Yes^a^	58	90.67	18.00	56	68.96	16.33	56	87.54	17.09
	Correlation ρ^b^		− 0.273*			− 0.175			0.118	
sub-1	None	6	93.67	14.17	6	68.50	20.19	6	80.83	20.52
	Yes^a^	35	86.29	16.70	33	65.44	15.57	33	86.91	18.61
	Correlation ρ^b^		− 0.169			− 0.105			0.130	
sub-2	None	6	97.67	15.82	6	63.00	17.04	6	72.00	15.06
	Yes^a^	18	79.78	15.44	17	60.25	10.76	17	80.78	14.24
	Correlation ρ^b^		− 0.666**			− 0.075			0.373	
sub-3	None	4	101.75	17.95	4	58.75	19.07	4	65.25	12.95
	Yes^a^	21	88.57	19.79	20	59.64	14.63	20	75.66	15.78
	Correlation ρ^b^		− 0.344			− 0.119			0.301	
sub-4	None	4	94.75	11.18	3	83.33	6.66	3	103.00	8.19
	Yes^a^	10	104.40	13.83	10	86.88	13.55	10	100.92	7.59
	Correlation ρ^b^		0.186			− 0.250			− 0.153	

Some patients were assigned to several subgroups

sub-1: Pat. with concomitant cardiac diseases; sub-2: Pat. with obstructive ventilatory disorder; sub-3: Pat. with lung function pattern indicating pulmonary emphysema; sub-4: Pat. without other pulmonary or cardiac diseases

*VC* vital capacity, *D*_LCO_ diffusion capacity for CO, *D*_LCO_/*VA* D_LCO_ in relation to alveolar volume, %*pred*. % of the respective predicted value, *SD* standard deviation

^*^Significant (*P* < 0.05)

^**^Significant (*P* < 0.01)

^a^Indicates pleural plaque score (Score B) ≥ 1

^b^Correlation ρ of pleural plaques (Score B) with lung function parameters

Overall, D_LCO_/VA is in the pathological range (< LLN) in only 9 of the patients studied. Accuracy with the finding of pleural plaques is low at 23%, and Cohen's κ is "poor" (κ = − 0.05). In the overall cohort, there is no correlation between D_LCO_/VA and pleural plaque score (ρ = 0.118, n.s.). Only patients with no other pulmonary or cardiac diseases (sub-4) show a slight negative correlation between the extent of radiological pleural findings and the transfer coefficient (ρ = − 0.153, n.s.) (Table 5).

Subjects with pleural plaques as well as fibrosis show significant correlations between the radiological findings and both VC and D_LCO_ (ρ = − 0.313, − 0.292, resp., both *P* < 0.05). Correlation with D_LCO_/VA do not lead to meaningful results (ρ = 0.024, n.s.).

### Correlation of pulmonary function parameters considering the radiological parameters PB, SC, and RA (Score C)

**Fig. 3:**
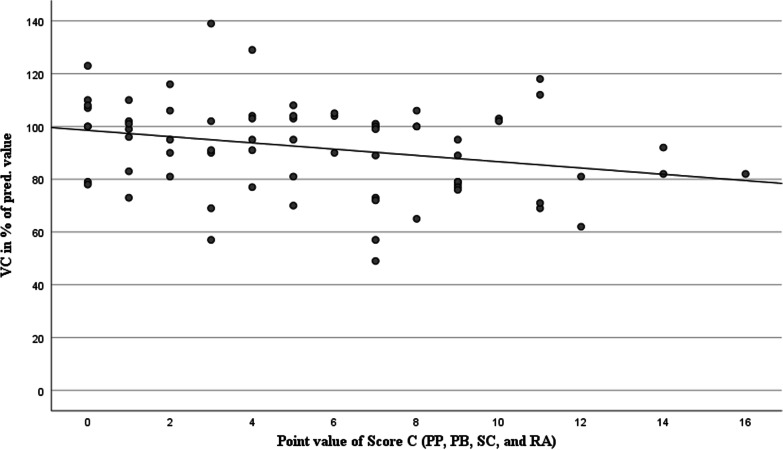
Comparison of vital capacity with the values of the newly quantified Score C. n = 72. ρ = -0.283, *P* < 0.05. PP: pleural plaques; PB: parenchymal bands; SC: subpleural curvilinear lines; RA: rounded atelectasis

**Fig. 4:**
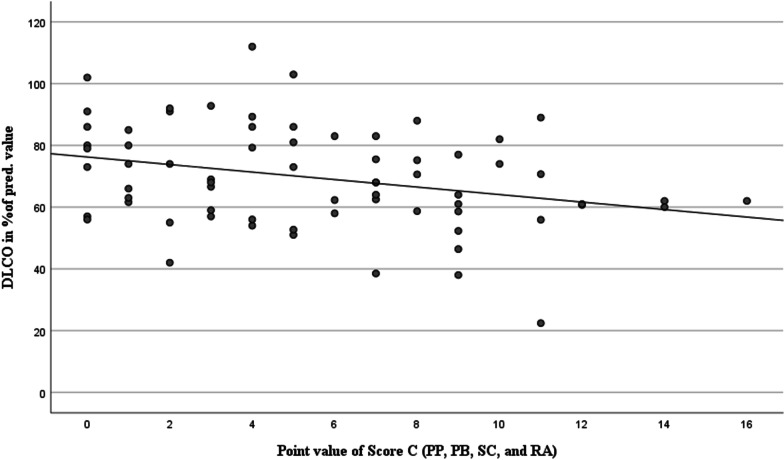
Comparison of diffusion capacity with the values of the newly quantified Score C. n = 69. ρ = -0.274, *P* < 0.05. PP: pleural plaques; PB: parenchymal bands; SC: subpleural curvilinear lines; RA: rounded atelectasis

Score C, newly developed in this study, includes the extent of pleural plaques as well as the extent of the newly evaluated and asbestos exposure-related radiological parameters PB, SC, and RA described above. Since effusion (EF) depicts a transient finding with only a temporary effect on pulmonary function, it was not included in the calculation of Score C. The score is now composed of the point value for the following pulmonary alterations: pleural plaques + (PB + SC + RA). The values determined for the patients in our study vary from 0–16 points (of possible 60 points). Significant relations to the new Score C are shown for both VC and D_LCO_: The correlation of this score with vital capacity is most distinct compared to both Scores A and B (ρ = − 0.283, *P* < 0.05), but also the D_LCO_ shows a notably stronger relation to Score C with a correlation of ρ = − 0.274 (*P* < 0.05) than to Score B, which only considers the expression of pleural plaques (ρ = − 0.175, n.s.). This shows that the expression of pleural plaques as a sole asbestos-associated CT finding has a lower relation to gas exchange disturbance than is the case when the newly quantified parameters are added. Figures 3 and 4 show the correlation of VC and D_LCO_ (in % of the respective predicted value) with the newly developed Score C, which also considers the subpleural, parenchymal asbestos-associated alterations.

D_LCO_/VA again shows no relevance with a correlation of ρ = 0.024 (n.s.) to Score C. Analyses of the individual additional parameters reveal significant correlations between parenchymal bands (PB) and VC (ρ = − 0.234, *P* < 0.05) and between PB and D_LCO_ (ρ = − 0.308, *P* < 0.05).

## Discussion

Pulmonary function diagnostics and radiological assessment complement each other well in the early diagnostics of persons formerly exposed to asbestos. Pulmonary function testing is easy to perform, provides immediate results and allows conclusions to be drawn about the functional state as well as the performance of the lungs, which is essential for the patients’ daily life. CT allows the detection of structural and morphological alterations. Pleural plaques are a typical radiologic finding associated with asbestos [34]. In addition to pleural plaques, early-stage fibrosis was also frequently observed in the collective presented here (see Table 3). The aim of the study was to confirm the relationship between VC, D_LCO_, and the respective CT findings and to further analyse the predictive benefit of abnormal lung function values with regard to the presence of morphological structural alterations. Moreover, we investigated whether the quantification of radiologically visible asbestos-associated additional parameters yields a diagnostic benefit.

The correlations between lung function parameters and fibrosis and pleural plaque scores (Scores A and B) in our study show that VC is a good predictive parameter regarding the expression of pleural plaques (ρ = − 0.273, *P* < 0.05). D_LCO_ shows a strong relation to the extent of asbestos-related fibrosis (ρ = − 0.315, *P* < 0.01), confirming the results of Barnikel et al. [35]. Likewise, Manners et al. (2017) showed a strong correlation between D_LCO_ and radiologically visible expression of interstitial pulmonary fibrosis in an asbestos-exposed collective [36]. In the fourfold table, however, D_LCO_ shows higher accuracy and sensitivity regarding the presence of pleural plaques in our study compared to VC (accuracy 61%, 39%, resp.; sensitivity 66%, 26%, resp.). These results thus confirm the essential role of D_LCO_ measurement in the early detection of asbestos-related alterations in the lungs in addition to the established method of VC measurement [37]. Therefore, both measurement methods should be used in the context of control and preventive examinations of persons formerly exposed to asbestos. The supplementary measurement of D_LCO_/VA is useful at most in asbestos-exposed patients without further pulmonary or cardiac diseases but generally does not allow relevant conclusions to be drawn about morphological alterations in the lungs. This confirms the results of van der Lee et al. (2006); these authors also did not see any additional diagnostic benefit of the transfer coefficient in the detection of pulmonary disease and, in particular, interstitial lung disease [38].

Evaluation of Score C, which considers the radiological parameters PB, SC, and RA, shows the best relation to changes in VC out of the three scores (ρ = − 0.283, *P* < 0.05); there is also a significant correlation with D_LCO_ (ρ = − 0.274, *P* < 0.05). From the results, it can be seen that the addition and quantification of subpleural alterations PB, SC, and RA are essential for predicting and assessing lung function changes (especially gas exchange) compared with considering the expression of pleural plaques alone. This confirms the results of Sette et al. (2004), who found a significant association between the extent of abnormalities at CT and impairment of gas exchange, especially when several lesion types were found concomitantly [39].

Both pulmonary function testing and radiological imaging are important in a possible process for compensation in Germany. Many cases are not detected by pulmonary function testing, especially spirometry alone, thus the addition of a TCT is important. However, radiological evidence of lung changes alone does not imply a claim for compensation in Germany, this is only given in the case of functionally proven lung damage. The sole risk of deterioration or tumour formation is not covered under current law.

Another aim of the study was to assess the influence of pulmonary emphysema and other possible confounding factors on the relationship between changes in lung function (especially diffusing capacity) and previous asbestos exposure. Emphysema can cause an attenuation of the D_LCO_ [24]. When analysing individuals exposed to asbestos who also have emphysema, it is difficult to distinguish whether the reduced D_LCO_ value is caused by the asbestos-induced remodeling processes in the lung or by the emphysema. Therefore, the subgroup of subjects with emphysema was evaluated separately. This approach was confirmed by the results of the subgroup analysis, which show that D_LCO_ is worst in patients with emphysema (sub-3) (see Table 2). The study presented here also demonstrates a substantial worsening of D_LCO_ values in patients with concomitant cardiac diseases (sub-1). It can thus be assumed that reduced cardiac performance has a negative impact on D_LCO_ as well [40]. However, the groups sub-2 and sub-4 also included several patients with reduced D_LCO_. Therefore, it is postulated that in all subgroups, asbestos-related changes contribute to the limitations of D_LCO_, which is reflected in the negative correlation of D_LCO_ with Score C in the overall collective. A previous study, which excluded patients with concomitant diseases such as emphysema or heart failure, detected a significant reduction in D_LCO_ associated with inhalation of asbestos fibre dust [10].

Subgroup analysis shows that, especially in patients with obstructive ventilatory disorder, VC and D_LCO_ are well suited for the assessment of asbestos-related pleural and pulmonary alterations. In the groups with concomitant cardiac diseases (sub-1) and pulmonary emphysema (sub-3), VC and D_LCO_ are important components in the diagnostics, but the relations are statistically less clear due to the confounders mentioned above.

## Limitations and strengths

All TCTs were assessed by the same person (radiologist with subject matter expertise); in this, strengths and weaknesses lie close together. Thus, the findings were not reviewed by a second person. However, according to Suganuma et al. [41], the benefit of a second evaluation is rather small when using the ICOERD. In addition, potential bias due to interrater variability was avoided.

A strength of the study lies in the fulfilment of essential quality features with regard to technical acquisition of HR-TCT examinations (slice thickness, number of slices, dose, positioning, etc.). This ensured uniformly good image quality.

A possible weakness of the study is because many patients are smokers or ex-smokers. Long-term smoking may show changes in the CT image, which may be difficult to differentiate from mild asbestos-related fibrosis [42, 43]. However, data have also been published describing mainly fibrosis of the alveolar walls in smoking-related genesis, whereas signs of asbestosis may occur throughout the parenchyma [44].

In this retrospective evaluation, the patient collective was limited, so that the division of the collective into subgroups led to partly non-significant results and may preclude firm conclusions. In the subgroup analysis, it should be noted that some patients were assigned to more than one subgroup and not exclusively to one subgroup.

## Conclusions

The correlation between HR-TCT findings and lung function changes confirms the relevance of measuring VC and D_LCO_ for the early detection of functional and morphologic asbestos-associated alterations. Even the partly less extensive expression of asbestos-related changes in HR-TCT in our cohort nevertheless showed a strong relation to changes in lung function. Thus, low-dose HR-TCT represents an integral component of early detection, along with spirometry and determination of D_LCO_. The standardization of HR-TCT acquisition and evaluation according to ICOERD is essential. In addition to quantification of pleural plaques and fibrosis, subpleural changes (PB, SC, RA) are of particular importance in predicting potential functional limitations. The quantification of these additional ICOERD parameters presented here, as well as standard measurement of spirometry and D_LCO_, are recommended for improved secondary prevention of asbestos-related disease.

## Supplementary Information

**Additional file 1:** Raw data of VC, DLCO, and radiological findings in asbestos exposed workers.

**Additional file 2:** Data fourfold tables of all subjects and subgroups.

## Data Availability

All data generated or analysed during this study are included in this published article and its Additional files 1 and 2.

## Abbreviations

%pred.: Percent of the respective predicted value
AG DRauE: Working Group for Diagnostic Radiology in Occupational and Environmental Diseases
ATS: American Thoracic Society
BMI: Body Mass Index
CO: Carbon monoxide
Cohen’s κ: Cohen’s kappa coefficient
CT: Computed tomography
DKG: German Society of Cardiology
D_LCO_: CO diffusion capacity
D_LCO_/VA: Diffusion capacity with CO in relation to alveolar volume
ECSC: European Coal and Steel Community
EF: Pleural effusion, free or loculated
ERS: European Respiratory Society
FEV1: One-second forced expiratory volume
FEV1/FVC: One-second forced expiratory volume in relation to forced expiratory vital capacity
Fig.: Figure
FRC_pleth_: Functional residual capacity in body plethysmography
FVC: Forced expiratory vital capacity
GLI: Global Lung Initiative
Hb: Haemoglobin content of the blood
HR-TCT: High-resolution computed tomography of the chest
ICOERD: International Classification of Occupational and Environmental Respiratory Diseases
L: Lower field
LLN: Lower limit of normal
M: Middle field
Mean: Average (mean) value in a sample
Median: Median value of a range of values
n: Number (of subjects included)
n.s.: Not significant
p: Probability value
Pat.: Patients
PB: Parenchymal bands
PP: Pleural plaques
PPV: Positive predictive value
py: Pack years
RA: Rounded atelectasis
resp.: Respective
RV: Residual volume
RV/TLC: Share of residual volume in total lung capacity
SB: Single breath
SC: Subpleural curvilinear lines
SD: Standard deviation
sRt: Total specific respiratory resistance
sub: Subgroup
TCT: Computed tomography of the thorax
TLC: Total lung capacity
U: Upper field
ULN: Upper limit of normal
VA: Alveolar volume
VC: Vital capacity
VC_max_: Largest measured vital capacity
ρ: Spearman's rank correlation coefficient (Spearman's rho)
