# Supplementary material for: Restrictions of VC and DLCO in relation to asbestos-related computed tomographic findings quantified by ICOERD-based parameters
Source: BMC Pulm Med. 2022 Jun 20;22:236. doi: 10.1186/s12890-022-02022-x (PMC9208103; doi:10.1186/s12890-022-02022-x)
Supplement: Supplementary file 1 — Additional file 1: Raw data of VC, DLCO, and radiological findings in asbestos exposed workers. [file 12890_2022_2022_MOESM1_ESM.docx]

Restrictions of VC and DLCO in relation to asbestos-related computed tomographic findings quantified by ICOERD-based parameters

Raw Data of the 72 subjects

| No | Age | Sex (0=male) | | Weight (kg) | | Height (cm) | | BMI | | Packyears | | Hemoglobin | Profession | | |
| --- | --- | --- | --- | --- | --- | --- | --- | --- | --- | --- | --- | --- | --- | --- | --- |
| 1 | 83 | 0 | 87 | | 172 | | 29.40 | | 15.0 | | 13.9 | | | Electrician |  |
| 2 | 80 | 0 | 84 | | 180 | | 25.90 | | 5.0 | | 13.4 | | | Locksmith |  |
| 3 | 82 | 0 | 100 | | 175 | | 32.70 | | 30.0 | | 13.2 | | | Locksmith |  |
| 4 | 71 | 0 | 96 | | 160 | | 37.50 | | 15.0 | | 17.1 | | | Shipbuilder |  |
| 5 | 66 | 0 | 106 | | 179 | | 33.10 | | n.a. | | n.a. | | | Shipbuilder |  |
| 6 | 65 | 0 | 90 | | 175 | | 29.40 | | n.a. | | n.a. | | | Isolator |  |
| 7 | 60 | 0 | 68 | | 170 | | 23.50 | | 40.0 | | n.a. | | | Plumber |  |
| 8 | 62 | 0 | 69 | | 168 | | 24.40 | | 47.0 | | n.a. | | | Automotive mechanic |  |
| 9 | 71 | 0 | 81 | | 167 | | 29.00 | | n.a. | | 17.0 | | | Locksmith |  |
| 10 | 66 | 0 | 77 | | 174 | | 25.43 | | 46.0 | | 15.5 | | | Plumber |  |
| 11 | 77 | 0 | 85 | | 173 | | 28.40 | | 9.0 | | 16.8 | | | Electrician |  |
| 12 | 81 | 0 | 73 | | 180 | | 22.50 | | 0.0 | | 13.3 | | | Electro engineer |  |
| 13 | 77 | 0 | 77 | | 160 | | 30.10 | | 2.5 | | 16.8 | | | Locksmith |  |
| 14 | 67 | 0 | 94 | | 180 | | 29.00 | | 0.0 | | 15.7 | | | Shipbuilder |  |
| 15 | 76 | 0 | 96 | | 188 | | 27.20 | | 8.0 | | 14.2 | | | Dockworker |  |
| 16 | 51 | 0 | 89 | | 177 | | 28.40 | | 0.0 | | 14.8 | | | Engineer |  |
| 17 | 75 | 0 | 109 | | 186 | | 31.50 | | 30.0 | | 15.2 | | | Locksmith |  |
| 18 | 80 | 0 | 84 | | 170 | | 29.10 | | 60.0 | | 15.1 | | | Dockworker |  |
| 19 | 75 | 0 | 72 | | 178 | | 22.70 | | 60.0 | | 18.0 | | | Locksmith |  |
| 20 | 74 | 0 | 99 | | 179 | | 30.90 | | 25.0 | | 15.1 | | | Scaffolder |  |
| 21 | 75 | 0 | 103 | | 182 | | 31.10 | | 2.0 | | 15.4 | | | Carpenter |  |
| 22 | 68 | 0 | 87 | | 178 | | 27.50 | | 40.0 | | 14.2 | | | Isolator |  |
| 23 | 78 | 0 | 95 | | 182 | | 28.70 | | 30.0 | | 17.4 | | | n.a. |  |
| 24 | 55 | 0 | 119 | | 185 | | 34.80 | | 35.0 | | n.a. | | | Dockworker |  |
| 25 | 63 | 0 | 80 | | 173 | | 26.70 | | 45.0 | | n.a. | | | Automotive mechanic |  |
| 26 | 56 | 0 | 101 | | 173 | | 33.70 | | 30.0 | | n.a. | | | Locksmith |  |
| 27 | 73 | 0 | 82 | | 174 | | 27.10 | | 30.0 | | n.a. | | | Automotive mechanic |  |
| 28 | 59 | 0 | 73 | | 183 | | 21.80 | | 34.0 | | n.a. | | | Electro technician |  |
| 29 | 68 | 0 | 102 | | 170 | | 35.30 | | 53.0 | | 18.8 | | | Shipbuilder |  |
| 30 | 69 | 0 | 95 | | 168 | | 33.70 | | 0.0 | | 14.9 | | | Carpenter |  |
| 31 | 74 | 0 | 92 | | 166 | | 33.40 | | 46.0 | | 13.0 | | | Automotive mechanic |  |
| 32 | 68 | 0 | 68 | | 178 | | 21.50 | | 0.0 | | 15.0 | | | Electrician |  |
| 33 | 82 | 0 | 87 | | 167 | | 31.20 | | 30.0 | | 16.0 | | | Locksmith |  |
| 34 | 81 | 0 | 76 | | 173 | | 25.40 | | 40.0 | | 17.6 | | | Blacksmith |  |
| 35 | 71 | 0 | 109 | | 170 | | 37.70 | | 40.0 | | 13.3 | | | Automotive mechanic |  |
| 36 | 66 | 0 | 89 | | 176 | | 28.70 | | 52.0 | | 16.2 | | | Industrial clerk |  |
| 37 | 79 | 0 | 90 | | 165 | | 33.10 | | 2.0 | | n.a. | | | Electrician |  |
| 38 | 76 | 0 | 67 | | 170 | | 23.20 | | 25.0 | | 11.5 | | | Engineer |  |
| 39 | 75 | 0 | 65 | | 173 | | 21.70 | | 60.0 | | 11.8 | | | Carpenter |  |
| 40 | 63 | 0 | 84 | | 169 | | 29.40 | | 50.0 | | 18.1 | | | Isolator |  |
| 41 | 78 | 0 | 71 | | 175 | | 23.20 | | 10.0 | | 13.8 | | | Warehouse worker |  |
| 42 | 70 | 0 | 106 | | 175 | | 34.60 | | 0.0 | | 16.0 | | | Mechanical Engineer |  |
| 43 | 80 | 0 | 76 | | 173 | | 25.40 | | 31.0 | | 14.1 | | | Electrician |  |
| 44 | 70 | 0 | 70 | | 175 | | 22.90 | | 40.0 | | n.a. | | | Automotive mechanic |  |
| 45 | 62 | 0 | 93 | | 168 | | 33.00 | | 36.0 | | n.a. | | | Automotive mechanic |  |
| 46 | 56 | 0 | 125 | | 195 | | 32.90 | | 35.0 | | n.a. | | | Isolator |  |
| 47 | 75 | 0 | 110 | | 177 | | 19.00 | | 15.0 | | n.a. | | | Locksmith |  |
| 48 | 80 | 0 | 109 | | 187 | | 31.20 | | 15.0 | | n.a. | | | Locksmith |  |
| 49 | 58 | 0 | 81 | | 167 | | 29.00 | | 30.0 | | n.a. | | | Automotive mechanic |  |
| 50 | 62 | 0 | 93 | | 183 | | 27.80 | | 34.0 | | n.a. | | | Automotive mechanic |  |
| 51 | 68 | 0 | 77 | | 172 | | 26.00 | | 43.0 | | n.a. | | | Electrician |  |
| 52 | 68 | 0 | 96 | | 183 | | 28.70 | | 71.0 | | n.a. | | | Technician |  |
| 53 | 77 | 0 | 97 | | 190 | | 26.90 | | 34.0 | | n.a. | | | Engineer |  |
| 54 | 77 | 0 | 100 | | 176 | | 32.30 | | 30.0 | | n.a. | | | Electro technician |  |
| 55 | 59 | 0 | 95 | | 171 | | 32.50 | | 50.0 | | n.a. | | | Installer |  |
| 56 | 66 | 0 | 122 | | 176 | | 39.40 | | 50.0 | | n.a. | | | Locksmith |  |
| 57 | 78 | 0 | 72 | | 172 | | 24.30 | | 50.0 | | n.a. | | | n.a. |  |
| 58 | 60 | 0 | 103 | | 179 | | 32.10 | | 42.0 | | n.a. | | | Automotive mechanic |  |
| 59 | 79 | 0 | 69 | | 168 | | 24.40 | | 49.0 | | n.a. | | | Construction worker |  |
| 60 | 58 | 0 | 103 | | 197 | | 26.50 | | 38.0 | | n.a. | | | Locksmith |  |
| 61 | 64 | 0 | 87 | | 184 | | 25.70 | | 49.0 | | n.a. | | | Installer |  |
| 62 | 72 | 0 | 89 | | 169 | | 31.20 | | 4.0 | | n.a. | | | Installer |  |
| 63 | 71 | 0 | 81 | | 173 | | 27.06 | | 49.0 | | n.a. | | | n.a. |  |
| 64 | 58 | 0 | 120 | | 188 | | 34.00 | | 34.0 | | n.a. | | | Shipbuilder |  |
| 65 | 69 | 0 | 122 | | 175 | | 39.80 | | 30.0 | | n.a. | | | Isolator |  |
| 66 | 74 | 0 | 117 | | 169 | | 41.00 | | 50.0 | | n.a. | | | Painter |  |
| 67 | 67 | 0 | 56 | | 179 | | 17.50 | | 35.0 | | n.a. | | | Electro technician |  |
| 68 | 63 | 0 | 96 | | 183 | | 28.70 | | 42.0 | | n.a. | | | Monteur |  |
| 69 | 68 | 0 | 105 | | 170 | | 36.30 | | 50.0 | | n.a. | | | Carpenter |  |
| 70 | 85 | 0 | 76 | | 172 | | 25.70 | | 50.0 | | n.a. | | | Dockworker |  |
| 71 | 71 | 0 | 50 | | 162 | | 19.10 | | 55.0 | | n.a. | | | Electrician |  |
| 72 | 55 | 0 | 74 | | 176 | | 23.90 | | 33.0 | | n.a. | | | Isolator |  |

| No | SRt_ULN | SRt_pred | SRt_measured | SRt_%pred | TLC_LLN | TLC_ULN | TLC_pred | TLC_meas. | TLC_%pred |
| --- | --- | --- | --- | --- | --- | --- | --- | --- | --- |
| 1 | 1.21 | 1.18 | 0.32 | 32 | 5.51 | 7.81 | 6.66 | 5.73 | 86 |
| 2 | 1.21 | 1.18 | 2.98 | 354 | 6.15 | 8.45 | 7.30 | 6.07 | 83 |
| 3 | 1.21 | 1.18 | 1.18 | 100 | 5.75 | 8.05 | 6.90 | 5.58 | 81 |
| 4 | 1.21 | 1.18 | 1.35 | 115 | 4.56 | 6.85 | 5.70 | 4.29 | 75 |
| 5 | 1.21 | 1.18 | 2.36 | 201 | 6.07 | 8.37 | 7.22 | 6.64 | 92 |
| 6 | 1.21 | 1.18 | 1.26 | 107 | 5.75 | 8.05 | 6.90 | 6.37 | 92 |
| 7 | 1.21 | 1.18 | 1.55 | 131 | 5.35 | 7.65 | 6.50 | 8.27 | 127 |
| 8 | 1.21 | 1.18 | 0.86 | 73 | 5.20 | 7.49 | 6.34 | 9.45 | 149 |
| 9 | 1.21 | 1.18 | 0.44 | 38 | 5.12 | 7.41 | 6.26 | 5.91 | 94 |
| 10 | 1.21 | 1.18 | 0.60 | 51 | 5.67 | 7.97 | 6.82 | 6.14 | 90 |
| 11 | 1.21 | 1.18 | 0.70 | 59 | 5.59 | 7.89 | 6.74 | 6.57 | 97 |
| 12 | 1.21 | 1.18 | 0.42 | 36 | 6.15 | 8.45 | 7.30 | 8.16 | 112 |
| 13 | 1.21 | 1.18 | 0.60 | 51 | 4.56 | 6.85 | 5.70 | 4.50 | 79 |
| 14 | 1.21 | 1.18 | 0.48 | 41 | 6.15 | 8.45 | 7.30 | 7.89 | 108 |
| 15 | 1.21 | 1.18 | 0.88 | 75 | 6.79 | 9.09 | 7.94 | 9.24 | 116 |
| 16 | 1.21 | 1.18 | 0.37 | 31 | 5.91 | 8.21 | 7.06 | 8.13 | 115 |
| 17 | 1.21 | 1.18 | 0.56 | 48 | 6.63 | 8.93 | 7.78 | 7.31 | 94 |
| 18 | 1.21 | 1.18 | 0.80 | 68 | 5.35 | 7.65 | 6.50 | 7.11 | 109 |
| 19 | 1.21 | 1.18 | 1.39 | 118 | 5.99 | 8.29 | 7.14 | 6.93 | 97 |
| 20 | 1.21 | 1.18 | 1.24 | 105 | 6.07 | 8.37 | 7.22 | 8.17 | 113 |
| 21 | 1.21 | 1.18 | 1.69 | 143 | 6.31 | 8.61 | 7.46 | 8.22 | 110 |
| 22 | 1.21 | 1.18 | 4.05 | 344 | 5.99 | 8.29 | 7.14 | 5.08 | 71 |
| 23 | 1.21 | 1.18 | 0.55 | 47 | n.a. | 8.61 | 7.46 | 7.74 | 104 |
| 24 | 1.21 | 1.18 | 1.32 | 112 | 6.55 | 8.85 | 7.70 | 8.08 | 105 |
| 25 | 1.21 | 1.18 | 0.72 | 61 | 5.59 | 7.89 | 6.74 | 6.58 | 98 |
| 26 | 1.21 | 1.18 | 0.94 | 80 | 5.59 | 7.89 | 6.74 | 6.98 | 104 |
| 27 | 1.21 | 1.18 | 0.90 | 76 | 5.67 | 7.97 | 6.82 | 4.84 | 71 |
| 28 | 1.21 | 1.18 | 1.18 | 100 | 6.39 | 8.69 | 7.54 | 10.17 | 135 |
| 29 | 1.21 | 1.18 | 1.10 | 94 | 5.35 | 7.65 | 6.50 | 6.59 | 101 |
| 30 | 1.21 | 1.18 | 0.87 | 74 | 5.20 | 7.49 | 6.34 | 4.69 | 74 |
| 31 | 1.21 | 1.18 | 2.16 | 183 | 5.04 | 7.33 | 6.18 | 6.78 | 110 |
| 32 | 1.21 | 1.18 | 0.71 | 61 | 5.99 | 8.29 | 7.14 | 7.69 | 108 |
| 33 | 1.21 | 1.18 | 1.77 | 150 | 5.12 | 7.41 | 6.26 | 6.77 | 108 |
| 34 | 1.21 | 1.18 | 4.16 | 353 | 5.59 | 7.89 | 6.74 | 6.53 | 97 |
| 35 | 1.21 | 1.18 | 0.95 | 81 | 5.35 | 7.65 | 6.50 | 6.89 | 106 |
| 36 | 1.21 | 1.18 | 0.46 | 39 | 5.83 | 8.13 | 6.98 | 6.81 | 98 |
| 37 | 1.21 | 1.18 | 1.65 | 141 | 4.96 | 7.25 | 6.10 | 5.87 | 96 |
| 38 | 1.21 | 1.18 | 2.51 | 213 | 5.35 | 7.65 | 6.50 | 10.72 | 165 |
| 39 | 1.21 | 1.18 | 1.60 | 136 | 5.59 | 7.89 | 6.74 | 7.59 | 113 |
| 40 | 1.21 | 1.18 | 0.73 | 62 | 5.28 | 7.57 | 6.42 | 7.13 | 111 |
| 41 | 1.21 | 1.18 | 7.21 | 736 | 5.75 | 8.05 | 6.90 | 9.14 | 132 |
| 42 | 1.21 | 1.18 | 0.96 | 81 | 5.75 | 8.05 | 6.90 | 6.01 | 87 |
| 43 | 1.21 | 1.18 | 0.68 | 58 | 5.59 | 7.89 | 6.74 | 4.13 | 61 |
| 44 | 1.21 | 1.18 | 0.75 | 64 | 5.75 | 8.05 | 6.90 | 7.55 | 109 |
| 45 | 1.21 | 1.18 | 1.05 | 89 | 5.20 | 7.49 | 6.34 | 5.79 | 91 |
| 46 | 1.21 | 1.18 | 1.44 | 122 | 7.35 | 9.65 | 8.50 | 10.07 | 118 |
| 47 | 1.21 | 1.18 | 1.87 | 159 | 5.91 | 8.21 | 7.06 | 8.36 | 118 |
| 48 | 1.21 | 1.18 | 1.32 | 113 | 6.71 | 9.01 | 7.86 | 6.85 | 87 |
| 49 | 1.21 | 1.18 | 1.26 | 107 | 5.12 | 7.41 | 6.26 | 6.22 | 99 |
| 50 | 1.21 | 1.18 | 1.41 | 120 | 6.39 | 8.69 | 7.54 | 7.29 | 97 |
| 51 | 1.21 | 1.18 | 2.81 | 239 | 5.51 | 7.81 | 6.66 | 7.64 | 115 |
| 52 | 1.21 | 1.18 | 1.26 | 108 | 6.39 | 8.69 | 7.54 | 8.51 | 113 |
| 53 | 1.21 | 1.18 | 0.89 | 76 | 6.95 | 9.25 | 8.10 | 8.06 | 99 |
| 54 | 1.21 | 1.18 | 1.67 | 142 | 5.83 | 8.13 | 6.98 | 6.11 | 87 |
| 55 | 1.21 | 1.18 | 0.92 | 78 | 5.43 | 7.73 | 6.58 | 7.35 | 112 |
| 56 | 1.21 | 1.18 | 0.88 | 75 | 5.83 | 8.13 | 6.98 | 7.78 | 111 |
| 57 | 1.21 | 1.18 | 1.25 | 106 | 5.51 | 7.81 | 6.66 | 5.36 | 80 |
| 58 | 1.21 | 1.18 | 1.03 | 87 | 6.07 | 8.37 | 7.22 | 8.03 | 111 |
| 59 | 1.21 | 1.18 | 0.61 | 52 | 5.20 | 7.49 | 6.34 | 8.23 | 130 |
| 60 | 1.21 | 1.18 | 1.04 | 88 | 7.51 | 9.81 | 8.66 | 9.01 | 104 |
| 61 | 1.21 | 1.18 | 0.86 | 73 | 6.47 | 8.77 | 7.62 | 8.89 | 117 |
| 62 | 1.21 | 1.18 | 1.08 | 91 | 5.28 | 7.57 | 6.42 | 8.24 | 128 |
| 63 | 1.21 | 1.18 | 1.92 | 163 | 5.59 | 7.89 | 6.74 | 7.62 | 113 |
| 64 | 1.21 | 1.18 | 0.84 | 71 | 6.79 | 9.09 | 7.94 | 9.78 | 123 |
| 65 | 1.21 | 1.18 | 0.77 | 66 | 5.75 | 8.05 | 6.90 | 6.62 | 96 |
| 66 | 1.21 | 1.18 | 1.10 | 93 | 5.28 | 7.57 | 6.42 | 5.65 | 88 |
| 67 | 1.21 | 1.18 | 0.68 | 58 | 6.07 | 8.37 | 7.22 | 7.18 | 99 |
| 68 | 1.21 | 1.18 | 0.66 | 56 | 6.39 | 8.69 | 7.54 | 7.49 | 99 |
| 69 | 1.21 | 1.18 | 2.62 | 223 | 5.35 | 7.65 | 6.50 | 6.97 | 107 |
| 70 | 1.21 | 1.18 | 3.13 | 266 | 5.51 | 7.81 | 6.66 | 12.25 | 184 |
| 71 | 1.21 | 1.18 | 1.99 | 169 | 4.72 | 7.01 | 5.86 | 7.35 | 125 |
| 72 | 1.21 | 1.18 | 0.85 | 72 | 5.83 | 8.13 | 6.98 | 7.58 | 109 |

| No | FRCp_  LLN | FRCp_  ULN | FRCp_  pred | FRCp_  meas | FRCp_  %pred | RV_LLN | RV_ULN | RV_pred | RV_meas | RV_  %pred |
| --- | --- | --- | --- | --- | --- | --- | --- | --- | --- | --- |
| 1 | 2.70 | 4.67 | 3.68 | 2.57 | 70 | 2.18 | 3.52 | 2.85 | 2.20 | 77 |
| 2 | 3.95 | 5.19 | 4.57 | 3.62 | 79 | 2.22 | 3.56 | 2.89 | 3.57 | 124 |
| 3 | 2.76 | 4.73 | 3.74 | 3.78 | 101 | 2.19 | 3.54 | 2.87 | 3.14 | 110 |
| 4 | 2.31 | 4.28 | 3.29 | 2.38 | 72 | 1.76 | 3.10 | 2.43 | 1.81 | 74 |
| 5 | 2.71 | 4.68 | 3.69 | 4.43 | 120 | 1.89 | 3.24 | 2.57 | 3.41 | 133 |
| 6 | 2.61 | 4.57 | 3.59 | 3.70 | 103 | 1.82 | 3.16 | 2.49 | 3.10 | 124 |
| 7 | 2.44 | 4.41 | 3.43 | 4.92 | 143 | 1.64 | 2.99 | 2.32 | 4.63 | 200 |
| 8 | 2.42 | 4.38 | 3.40 | 5.48 | 161 | 1.66 | 3.01 | 2.33 | 4.72 | 202 |
| 9 | 2.43 | 4.40 | 3.41 | 3.19 | 94 | 1.74 | 3.08 | 2.41 | 2.64 | 109 |
| 10 | 2.59 | 4.56 | 3.58 | 4.01 | 112 | 1.83 | 3.17 | 2.50 | 3.14 | 125 |
| 11 | 2.67 | 4.64 | 3.65 | 3.31 | 91 | 2.06 | 3.40 | 2.73 | 2.73 | 100 |
| 12 | 2.87 | 4.84 | 3.85 | 4.34 | 113 | 2.24 | 3.58 | 2.91 | 4.09 | 140 |
| 13 | 2.36 | 4.33 | 3.35 | 2.55 | 76 | 1.89 | 3.23 | 2.56 | 1.86 | 73 |
| 14 | 2.74 | 4.71 | 3.72 | 4.03 | 108 | 1.93 | 3.27 | 2.60 | 3.79 | 146 |
| 15 | 3.01 | 4.98 | 3.99 | 4.60 | 115 | 2.23 | 3.58 | 2.90 | 4.40 | 151 |
| 16 | 2.53 | 4.49 | 3.51 | 4.06 | 116 | 1.54 | 2.88 | 2.21 | 3.34 | 151 |
| 17 | 2.95 | 4.92 | 3.94 | 3.30 | 84 | 2.18 | 3.53 | 2.86 | 3.11 | 109 |
| 18 | 2.62 | 4.59 | 3.61 | 4.12 | 114 | 2.08 | 3.43 | 2.76 | 3.36 | 122 |
| 19 | 2.77 | 4.73 | 3.75 | 5.26 | 140 | 2.08 | 3.42 | 2.75 | 2.33 | 85 |
| 20 | 2.78 | 4.75 | 3.76 | 3.95 | 105 | 2.07 | 3.42 | 2.74 | 3.91 | 142 |
| 21 | 2.86 | 4.83 | 3.84 | 3.69 | 96 | 2.13 | 3.48 | 2.80 | 3.06 | 109 |
| 22 | 2.70 | 4.67 | 3.69 | 3.28 | 89 | 1.93 | 3.27 | 2.60 | 2.42 | 93 |
| 23 | n.a. | 4.85 | 3.87 | 4.55 | 117 | n.a. | 3.54 | 2.87 | 3.48 | 121 |
| 24 | 2.75 | 4.72 | 3.73 | 4.03 | 108 | 1.73 | 3.08 | 2.40 | 3.83 | 159 |
| 25 | 2.54 | 4.51 | 3.53 | 3.71 | 105 | 1.75 | 3.09 | 2.42 | 2.66 | 110 |
| 26 | 2.48 | 4.45 | 3.46 | 2.85 | 82 | 1.60 | 2.94 | 2.27 | 2.65 | 117 |
| 27 | 2.65 | 4.62 | 3.64 | 3.22 | 89 | 1.98 | 3.33 | 2.66 | 2.14 | 81 |
| 28 | 2.74 | 4.71 | 3.72 | 6.15 | 165 | 1.79 | 3.14 | 2.47 | 5.57 | 226 |
| 29 | 2.52 | 4.48 | 3.50 | 2.70 | 77 | 1.82 | 3.17 | 2.49 | 2.62 | 105 |
| 30 | 2.48 | 4.45 | 3.46 | 2.74 | 79 | 1.82 | 3.16 | 2.49 | 2.16 | 87 |
| 31 | 2.48 | 4.44 | 3.46 | 4.50 | 130 | 1.90 | 3.24 | 2.57 | 4.09 | 159 |
| 32 | 2.72 | 4.69 | 3.71 | 5.51 | 149 | 1.97 | 3.31 | 2.64 | 2.77 | 105 |
| 33 | 2.57 | 4.54 | 3.56 | 4.29 | 121 | 2.09 | 3.43 | 2.76 | 4.11 | 149 |
| 34 | 2.70 | 4.67 | 3.69 | 3.95 | 107 | 2.15 | 3.49 | 2.82 | 3.94 | 140 |
| 35 | 2.54 | 4.51 | 3.53 | 3.30 | 94 | 1.89 | 3.23 | 2.56 | 3.09 | 121 |
| 36 | 2.64 | 4.61 | 3.62 | 3.15 | 87 | 1.86 | 3.20 | 2.53 | 2.79 | 110 |
| 37 | 2.50 | 4.47 | 3.48 | 3.78 | 109 | 2.00 | 3.34 | 2.67 | 3.27 | 122 |
| 38 | 2.59 | 4.56 | 3.57 | 3.50 | 98 | 2.00 | 3.34 | 2.67 | 7.96 | 298 |
| 39 | 2.65 | 4.62 | 3.63 | 5.31 | 146 | 2.01 | 3.36 | 2.69 | 4.93 | 184 |
| 40 | 2.45 | 4.42 | 3.43 | 4.40 | 128 | 1.70 | 3.04 | 2.37 | 3.61 | 152 |
| 41 | 2.72 | 4.69 | 3.71 | 8.07 | 218 | 2.11 | 3.45 | 2.78 | 6.96 | 250 |
| 42 | 2.65 | 4.62 | 3.63 | 2.79 | 77 | 1.93 | 3.27 | 2.60 | 2.56 | 98 |
| 43 | 2.69 | 4.66 | 3.68 | 3.38 | 92 | 2.12 | 3.47 | 2.80 | 2.32 | 83 |
| 44 | 2.65 | 4.62 | 3.63 | 4.03 | 111 | 1.93 | 3.27 | 2.60 | 3.69 | 142 |
| 45 | 2.42 | 4.38 | 3.40 | 3.28 | 97 | 1.66 | 3.01 | 2.33 | 2.75 | 118 |
| 46 | 2.99 | 4.96 | 3.98 | 5.53 | 139 | 1.88 | 3.23 | 2.56 | 4.03 | 158 |
| 47 | 2.74 | 4.71 | 3.73 | 4.43 | 119 | 2.07 | 3.41 | 2.74 | 4.17 | 152 |
| 48 | 3.01 | 4.98 | 3.99 | 4.33 | 108 | 2.31 | 3.65 | 2.98 | 0.22 | 7 |
| 49 | 2.36 | 4.32 | 3.34 | 2.68 | 80 | 1.56 | 2.91 | 2.23 | 2.31 | 103 |
| 50 | 2.77 | 4.73 | 3.75 | 3.69 | 98 | 1.86 | 3.20 | 2.53 | 2.54 | 100 |
| 51 | 2.56 | 4.53 | 3.55 | 5.68 | 160 | 1.85 | 3.19 | 2.52 | 4.11 | 163 |
| 52 | 2.82 | 4.79 | 3.80 | 5.05 | 133 | 1.99 | 3.34 | 2.66 | 3.58 | 134 |
| 53 | 3.06 | 5.03 | 4.05 | 4.87 | 120 | 2.28 | 3.63 | 2.95 | 4.31 | 146 |
| 54 | 2.74 | 4.71 | 3.72 | 3.52 | 95 | 2.10 | 3.44 | 2.77 | 3.27 | 118 |
| 55 | 2.46 | 4.43 | 3.44 | 3.71 | 108 | 1.64 | 2.98 | 2.31 | 2.80 | 121 |
| 56 | 2.64 | 4.61 | 3.62 | 4.08 | 113 | 1.86 | 3.20 | 2.53 | 3.45 | 137 |
| 57 | 2.65 | 4.62 | 3.64 | 3.30 | 91 | 2.07 | 3.41 | 2.74 | 2.35 | 86 |
| 58 | 2.65 | 4.62 | 3.64 | 3.90 | 107 | 1.76 | 3.11 | 2.43 | 3.40 | 140 |
| 59 | 2.57 | 4.54 | 3.55 | 5.21 | 147 | 2.04 | 3.38 | 2.71 | 3.52 | 130 |
| 60 | 3.06 | 5.03 | 4.04 | 4.02 | 100 | 1.95 | 3.30 | 2.63 | 3.27 | 124 |
| 61 | 2.81 | 4.78 | 3.79 | 5.42 | 143 | 1.92 | 3.26 | 2.59 | 4.31 | 166 |
| 62 | 2.53 | 4.50 | 3.51 | 3.88 | 111 | 1.90 | 3.24 | 2.57 | 3.54 | 138 |
| 63 | 2.61 | 4.58 | 3.60 | 4.59 | 128 | 1.93 | 3.27 | 2.60 | 4.43 | 170 |
| 64 | 2.85 | 4.82 | 3.83 | 4.75 | 124 | 1.84 | 3.18 | 2.51 | 4.33 | 173 |
| 65 | 2.64 | 4.61 | 3.63 | 2.70 | 74 | 1.91 | 3.25 | 2.58 | 2.40 | 93 |
| 66 | 2.55 | 4.51 | 3.53 | 3.36 | 95 | 1.94 | 3.28 | 2.61 | 2.72 | 104 |
| 67 | 2.72 | 4.69 | 3.70 | 3.64 | 98 | 1.92 | 3.26 | 2.59 | 3.17 | 123 |
| 68 | 2.78 | 4.74 | 3.76 | 3.29 | 88 | 1.88 | 3.23 | 2.55 | 2.53 | 99 |
| 69 | 2.52 | 4.48 | 3.50 | 4.72 | 135 | 1.82 | 3.17 | 2.49 | 4.04 | 162 |
| 70 | 2.72 | 4.68 | 3.70 | 9.54 | 258 | 2.22 | 3.57 | 2.89 | 8.45 | 292 |
| 71 | 2.36 | 4.32 | 3.34 | 4.27 | 128 | 1.78 | 3.13 | 2.45 | 3.92 | 160 |
| 72 | 2.54 | 4.51 | 3.52 | 3.14 | 89 | 1.61 | 2.96 | 2.29 | 2.65 | 116 |

| No | RV/TLC_  LLN | RV/TLC_ULN | RV/TLC_  pred | RV/TLC_meas | RV/TLC_%pred | VCmax_  LLN | VCmax_  ULN | VCmax_  pred | VCmax_meas | VCmax_%pred |
| --- | --- | --- | --- | --- | --- | --- | --- | --- | --- | --- |
| 1 | 37.38 | 55.28 | 46.33 | 38.42 | 83 | 2.60 | 4.44 | 3.52 | 3.53 | 100 |
| 2 | 36.21 | 54.11 | 45.16 | 58.85 | 130 | 3.35 | 5.45 | 4.40 | 2.50 | 57 |
| 3 | 36.99 | 54.89 | 45.94 | 56.24 | 122 | 2.81 | 4.65 | 3.73 | 2.44 | 65 |
| 4 | 32.70 | 50.60 | 41.65 | 42.17 | 101 | 2.20 | 4.04 | 3.12 | 2.48 | 79 |
| 5 | 30.75 | 48.65 | 39.70 | 51.32 | 129 | 3.50 | 5.34 | 4.42 | 3.23 | 73 |
| 6 | 30.36 | 48.26 | 39.31 | 48.64 | 124 | 3.29 | 5.12 | 4.20 | 3.27 | 78 |
| 7 | 28.41 | 46.31 | 37.36 | 56.04 | 150 | 4.96 | 3.12 | 4.04 | 3.63 | 90 |
| 8 | 29.19 | 47.09 | 38.14 | 49.89 | 131 | 2.94 | 4.78 | 3.86 | 4.74 | 123 |
| 9 | 30.75 | 48.65 | 39.70 | 44.59 | 112 | 2.77 | 4.61 | 3.69 | 3.28 | 89 |
| 10 | 30.75 | 48.65 | 39.70 | 51.09 | 129 | 3.20 | 5.03 | 4.12 | 3.00 | 73 |
| 11 | 35.04 | 52.94 | 43.99 | 41.52 | 94 | 2.83 | 4.67 | 3.75 | 3.84 | 103 |
| 12 | 36.60 | 54.50 | 45.55 | 50.03 | 110 | 3.14 | 4.98 | 4.06 | 4.08 | 100 |
| 13 | 35.04 | 52.94 | 43.99 | 41.27 | 94 | 2.04 | 3.87 | 2.95 | 2.64 | 89 |
| 14 | 31.14 | 49.04 | 40.09 | 48.09 | 120 | 3.54 | 5.37 | 4.45 | 4.10 | 92 |
| 15 | 34.65 | 52.55 | 43.60 | 47.55 | 109 | 3.77 | 5.61 | 4.69 | 4.85 | 103 |
| 16 | 24.90 | 42.80 | 33.85 | 41.08 | 121 | 3.80 | 5.64 | 4.72 | 4.79 | 101 |
| 17 | 34.26 | 52.16 | 43.21 | 42.55 | 98 | 3.68 | 5.51 | 4.60 | 4.20 | 91 |
| 18 | 36.21 | 54.11 | 45.16 | 47.20 | 105 | 2.56 | 4.40 | 3.48 | 3.76 | 108 |
| 19 | 34.26 | 52.16 | 43.21 | 33.66 | 78 | 3.19 | 5.03 | 4.11 | 4.60 | 112 |
| 20 | 33.87 | 51.77 | 42.82 | 47.82 | 112 | 3.28 | 5.12 | 4.20 | 4.26 | 102 |
| 21 | 34.26 | 52.16 | 43.21 | 37.28 | 86 | 3.43 | 5.27 | 4.35 | 5.16 | 118 |
| 22 | 31.53 | 49.43 | 40.48 | 47.53 | 117 | 3.39 | 5.22 | 4.30 | 2.67 | 62 |
| 23 | n.a. | 53.33 | 44.38 | 44.95 | 101 | 3.35 | 5.19 | 4.27 | 4.26 | 100 |
| 24 | 26.46 | 44.36 | 35.41 | 47.35 | 134 | 4.18 | 6.01 | 5.09 | 4.25 | 83 |
| 25 | 29.58 | 47.48 | 38.53 | 40.46 | 105 | 3.22 | 5.06 | 4.14 | 3.92 | 95 |
| 26 | 26.85 | 44.75 | 35.80 | 37.92 | 106 | 3.42 | 5.25 | 4.34 | 4.34 | 100 |
| 27 | 33.48 | 51.38 | 42.43 | 44.21 | 104 | 3.00 | 4.84 | 3.92 | 2.70 | 69 |
| 28 | 28.02 | 45.92 | 36.97 | 54.81 | 148 | 3.94 | 5.78 | 4.86 | 4.59 | 95 |
| 29 | 31.53 | 49.43 | 40.48 | 39.78 | 98 | 2.90 | 4.73 | 3.82 | 3.97 | 104 |
| 30 | 31.92 | 49.82 | 40.87 | 46.14 | 113 | 2.75 | 4.58 | 3.67 | 2.53 | 69 |
| 31 | 33.87 | 51.77 | 42.82 | 60.32 | 141 | 2.49 | 4.32 | 3.40 | 2.69 | 79 |
| 32 | 32.31 | 50.21 | 41.26 | 35.98 | 87 | 3.33 | 5.17 | 4.25 | 4.92 | 116 |
| 33 | 36.99 | 54.89 | 45.94 | 60.71 | 132 | 2.32 | 4.16 | 3.24 | 2.66 | 82 |
| 34 | 36.60 | 54.50 | 45.55 | 60.31 | 132 | 2.72 | 4.55 | 3.63 | 2.59 | 71 |
| 35 | 32.70 | 50.60 | 41.65 | 44.91 | 108 | 2.81 | 4.65 | 3.73 | 3.80 | 102 |
| 36 | 30.75 | 48.65 | 39.70 | 40.90 | 103 | 3.32 | 5.16 | 4.24 | 4.03 | 95 |
| 37 | 35.82 | 53.72 | 44.77 | 55.72 | 124 | 2.28 | 4.12 | 3.20 | 2.60 | 81 |
| 38 | 34.65 | 52.55 | 43.60 | 74.24 | 170 | 2.67 | 4.51 | 3.59 | 2.76 | 77 |
| 39 | 34.26 | 52.16 | 43.21 | 64.95 | 150 | 2.88 | 4.72 | 3.80 | 2.66 | 70 |
| 40 | 29.58 | 47.48 | 38.53 | 50.62 | 131 | 2.98 | 4.81 | 3.89 | 3.52 | 90 |
| 41 | 35.43 | 53.33 | 44.38 | 76.15 | 172 | 2.92 | 4.76 | 3.84 | 2.18 | 57 |
| 42 | 32.31 | 50.21 | 41.26 | 42.65 | 103 | 3.15 | 4.98 | 4.07 | 3.08 | 76 |
| 43 | 36.21 | 54.11 | 45.16 | 56.11 | 124 | 2.74 | 4.58 | 3.66 | 1.81 | 49 |
| 44 | 32.31 | 50.21 | 41.26 | 48.97 | 119 | 3.15 | 4.98 | 4.07 | 3.85 | 95 |
| 45 | 29.19 | 47.09 | 38.14 | 47.43 | 124 | 2.94 | 4.78 | 3.86 | 3.04 | 79 |
| 46 | 26.85 | 44.75 | 35.80 | 40.05 | 112 | 4.76 | 6.60 | 5.68 | 6.04 | 106 |
| 47 | 34.26 | 52.16 | 43.21 | 49.90 | 115 | 3.13 | 4.97 | 4.05 | 4.19 | 104 |
| 48 | 36.21 | 54.11 | 45.16 | 44.70 | 99 | 3.60 | 5.44 | 4.52 | 4.48 | 99 |
| 49 | 27.63 | 45.53 | 36.58 | 37.06 | 101 | 2.99 | 4.83 | 3.91 | 3.92 | 100 |
| 50 | 29.19 | 47.09 | 38.14 | 34.87 | 91 | 3.86 | 5.70 | 4.78 | 4.75 | 99 |
| 51 | 31.53 | 49.43 | 40.48 | 53.82 | 133 | 3.02 | 4.86 | 3.94 | 3.53 | 90 |
| 52 | 31.53 | 49.43 | 40.48 | 42.08 | 104 | 3.69 | 5.53 | 4.61 | 4.93 | 107 |
| 53 | 35.04 | 52.94 | 43.99 | 53.50 | 122 | 3.87 | 5.70 | 4.78 | 3.75 | 78 |
| 54 | 35.04 | 52.94 | 43.99 | 53.49 | 122 | 3.01 | 4.85 | 3.93 | 2.84 | 72 |
| 55 | 28.02 | 45.92 | 36.97 | 38.05 | 103 | 3.21 | 5.05 | 4.13 | 4.55 | 110 |
| 56 | 30.75 | 48.65 | 39.70 | 44.38 | 112 | 3.32 | 5.16 | 4.24 | 4.33 | 102 |
| 57 | 35.43 | 53.33 | 44.38 | 43.74 | 99 | 2.74 | 4.58 | 3.66 | 3.02 | 82 |
| 58 | 28.41 | 46.31 | 37.36 | 42.32 | 113 | 3.67 | 5.51 | 4.59 | 4.63 | 101 |
| 59 | 35.82 | 53.72 | 44.77 | 42.80 | 96 | 2.47 | 4.30 | 3.39 | 4.70 | 139 |
| 60 | 27.63 | 45.53 | 36.58 | 36.25 | 99 | 4.82 | 6.66 | 5.74 | 5.75 | 100 |
| 61 | 29.97 | 47.87 | 38.92 | 48.48 | 125 | 3.86 | 5.70 | 4.78 | 4.58 | 96 |
| 62 | 33.09 | 50.99 | 42.04 | 42.95 | 102 | 2.72 | 4.56 | 3.64 | 4.70 | 129 |
| 63 | 32.70 | 50.60 | 41.65 | 58.13 | 140 | 3.00 | 4.83 | 3.91 | 3.19 | 81 |
| 64 | 27.63 | 45.53 | 38.58 | 44.29 | 121 | 4.28 | 6.11 | 5.19 | 5.45 | 105 |
| 65 | 31.92 | 49.82 | 40.87 | 36.22 | 89 | 3.17 | 5.01 | 4.09 | 4.22 | 103 |
| 66 | 33.87 | 51.77 | 42.82 | 48.26 | 113 | 2.67 | 4.51 | 3.59 | 2.92 | 81 |
| 67 | 31.14 | 49.04 | 40.09 | 44.22 | 110 | 3.47 | 5.31 | 4.39 | 4.00 | 91 |
| 68 | 29.58 | 47.48 | 38.53 | 33.79 | 88 | 3.83 | 5.67 | 4.75 | 4.96 | 104 |
| 69 | 31.53 | 49.43 | 40.48 | 57.91 | 143 | 2.90 | 4.73 | 3.82 | 2.94 | 77 |
| 70 | 38.16 | 56.06 | 47.11 | 68.95 | 146 | 2.54 | 4.38 | 3.46 | 3.80 | 110 |
| 71 | 32.70 | 50.60 | 41.65 | 53.34 | 128 | 2.33 | 4.16 | 3.24 | 3.43 | 106 |
| 72 | 26.46 | 44.36 | 35.41 | 35.04 | 99 | 3.63 | 5.46 | 4.55 | 4.92 | 108 |

| No | VCin_LLN | VCin_  ULN | VCin_  pred | VCin_  meas | VCin_  %pred | FVC_LLN | FVC_ULN | FVC_pred | FVC_  meas | FVC_  %pred |
| --- | --- | --- | --- | --- | --- | --- | --- | --- | --- | --- |
| 1 | 2.60 | 4.44 | 3.52 | 3.53 | 100 | 2.49 | 4.50 | 3.50 | 3.23 | 92 |
| 2 | 3.35 | 5.45 | 4.40 | 2.50 | 57 | 3.35 | 5.45 | 4.40 | 2.28 | 52 |
| 3 | 2.81 | 4.65 | 3.73 | 2.44 | 65 | 2.62 | 4.72 | 3.67 | 2.27 | 62 |
| 4 | 2.20 | 4.04 | 3.12 | 2.41 | 77 | 2.39 | 4.10 | 3.25 | 2.48 | 76 |
| 5 | 3.50 | 5.34 | 4.42 | 2.90 | 66 | 3.32 | 5.57 | 4.45 | 3.23 | 73 |
| 6 | 3.29 | 5.12 | 4.20 | 3.06 | 73 | 3.18 | 5.30 | 4.24 | 3.27 | 77 |
| 7 | 4.96 | 3.12 | 4.04 | 3.62 | 89 | 3.16 | 5.15 | 4.15 | 3.63 | 88 |
| 8 | 2.94 | 4.78 | 3.86 | 4.55 | 118 | 2.98 | 4.92 | 3.95 | 4.61 | 117 |
| 9 | 2.77 | 4.61 | 3.69 | 3.12 | 85 | 2.82 | 4.72 | 3.77 | 3.28 | 87 |
| 10 | 3.20 | 5.03 | 4.12 | 3.00 | 73 | 3.12 | 5.22 | 4.17 | 2.97 | 71 |
| 11 | 2.83 | 4.67 | 3.75 | 3.84 | 103 | 2.71 | 4.76 | 3.73 | 3.70 | 99 |
| 12 | 3.14 | 4.98 | 4.06 | 3.81 | 94 | 2.84 | 5.08 | 3.96 | 3.92 | 99 |
| 13 | 2.04 | 3.87 | 2.95 | 2.57 | 87 | 2.23 | 3.93 | 3.08 | 2.64 | 86 |
| 14 | 3.54 | 5.37 | 4.45 | 4.03 | 91 | 3.36 | 5.64 | 4.50 | 4.09 | 91 |
| 15 | 3.77 | 5.61 | 4.69 | 4.80 | 102 | 3.34 | 5.85 | 4.59 | 4.85 | 106 |
| 16 | 3.80 | 5.64 | 4.72 | 1.87 | 40 | 3.80 | 5.98 | 4.89 | 4.79 | 98 |
| 17 | 3.68 | 5.51 | 4.60 | 4.20 | 91 | 3.31 | 5.76 | 4.53 | 4.15 | 92 |
| 18 | 2.56 | 4.40 | 3.48 | 3.76 | 108 | 2.51 | 4.47 | 3.49 | 3.72 | 107 |
| 19 | 3.19 | 5.03 | 4.11 | 4.39 | 107 | 2.96 | 5.16 | 4.06 | 4.60 | 113 |
| 20 | 3.28 | 5.12 | 4.20 | 4.13 | 98 | 3.04 | 5.27 | 4.16 | 4.07 | 98 |
| 21 | 3.43 | 5.27 | 4.35 | 4.86 | 112 | 3.11 | 5.43 | 4.27 | 5.05 | 118 |
| 22 | 3.39 | 5.22 | 4.30 | 2.67 | 62 | 3.20 | 5.42 | 4.31 | 2.49 | 58 |
| 23 | 3.35 | n.a. | 4.27 | 4.26 | 100 | 3.00 | n.a. | 4.16 | 4.10 | 99 |
| 24 | 4.18 | 6.01 | 5.09 | 3.93 | 77 | 4.06 | 6.50 | 5.28 | 4.25 | 81 |
| 25 | 3.22 | 5.06 | 4.14 | 3.92 | 95 | 3.20 | 5.27 | 4.24 | 3.73 | 88 |
| 26 | 3.42 | 5.25 | 4.34 | 4.27 | 98 | 3.45 | 5.52 | 4.48 | 4.34 | 97 |
| 27 | 3.00 | 4.84 | 3.92 | 2.70 | 69 | 2.87 | 4.96 | 3.92 | 2.47 | 63 |
| 28 | 3.94 | 5.78 | 4.86 | 4.59 | 95 | 3.83 | 6.21 | 5.02 | 4.53 | 90 |
| 29 | 2.90 | 4.73 | 3.82 | 3.90 | 102 | 2.88 | 4.86 | 3.87 | 3.85 | 99 |
| 30 | 2.75 | 4.58 | 3.67 | 2.15 | 59 | 2.75 | 4.68 | 3.72 | 2.53 | 68 |
| 31 | 2.49 | 4.32 | 3.40 | 2.37 | 70 | 2.54 | 4.41 | 3.47 | 2.31 | 67 |
| 32 | 3.33 | 5.17 | 4.25 | 4.92 | 116 | 3.14 | 5.35 | 4.24 | 4.91 | 116 |
| 33 | 2.32 | 4.16 | 3.24 | 2.65 | 82 | 2.35 | 4.23 | 3.29 | 2.48 | 75 |
| 34 | 2.72 | 4.55 | 3.63 | 2.56 | 70 | 2.56 | 4.61 | 3.59 | 2.47 | 69 |
| 35 | 2.81 | 4.65 | 3.73 | 3.66 | 98 | 2.78 | 4.76 | 3.77 | 3.80 | 101 |
| 36 | 3.32 | 5.16 | 4.24 | 3.83 | 90 | 3.22 | 5.37 | 4.29 | 3.85 | 90 |
| 37 | 2.28 | 4.12 | 3.20 | 2.59 | 81 | 2.34 | 4.16 | 3.25 | 2.42 | 75 |
| 38 | 2.67 | 4.51 | 3.59 | 2.62 | 73 | 2.62 | 4.59 | 3.61 | 2.76 | 77 |
| 39 | 2.88 | 4.72 | 3.80 | 2.66 | 70 | 2.77 | 4.82 | 3.80 | 2.32 | 61 |
| 40 | 2.98 | 4.81 | 3.89 | 3.18 | 82 | 3.02 | 4.98 | 4.00 | 3.30 | 83 |
| 41 | 2.92 | 4.76 | 3.84 | 1.84 | 48 | 2.73 | 4.84 | 3.79 | 1.91 | 51 |
| 42 | 3.15 | 4.98 | 4.07 | 3.08 | 76 | 3.00 | 5.12 | 4.06 | 3.45 | 85 |
| 43 | 2.74 | 4.58 | 3.66 | 1.40 | 38 | 2.61 | 4.66 | 3.64 | 1.81 | 50 |
| 44 | 3.15 | 4.98 | 4.07 | 3.85 | 95 | 3.03 | 5.15 | 4.09 | 3.62 | 88 |
| 45 | 2.94 | 4.78 | 3.86 | 2.83 | 73 | 3.00 | 4.94 | 3.97 | 3.00 | 76 |
| 46 | 4.76 | 6.60 | 5.68 | 5.86 | 103 | 4.59 | 7.36 | 5.97 | 6.04 | 101 |
| 47 | 3.13 | 4.97 | 4.05 | 4.19 | 104 | 2.90 | 5.07 | 3.99 | 3.91 | 98 |
| 48 | 3.60 | 5.44 | 4.52 | 4.04 | 89 | 3.16 | 5.63 | 4.40 | 4.48 | 102 |
| 49 | 2.99 | 4.83 | 3.91 | 3.88 | 99 | 3.09 | 4.99 | 4.04 | 3.92 | 97 |
| 50 | 3.86 | 5.70 | 4.78 | 4.42 | 92 | 3.70 | 6.07 | 4.88 | 4.75 | 97 |
| 51 | 3.02 | 4.86 | 3.94 | 3.05 | 78 | 2.96 | 5.00 | 3.98 | 3.53 | 89 |
| 52 | 3.69 | 5.53 | 4.61 | 4.70 | 102 | 3.45 | 5.82 | 4.64 | 4.93 | 106 |
| 53 | 3.87 | 5.70 | 4.78 | 3.48 | 73 | 3.37 | 5.94 | 4.66 | 3.70 | 80 |
| 54 | 3.01 | 4.85 | 3.93 | 2.60 | 66 | 2.82 | 4.96 | 3.89 | 2.64 | 68 |
| 55 | 3.21 | 5.05 | 4.13 | 4.19 | 102 | 3.23 | 5.25 | 4.24 | 4.55 | 107 |
| 56 | 3.32 | 5.16 | 4.24 | 4.33 | 102 | 3.18 | 5.34 | 4.26 | 4.04 | 95 |
| 57 | 2.74 | 4.58 | 3.66 | 3.02 | 82 | 2.64 | 4.66 | 3.65 | 3.00 | 82 |
| 58 | 3.67 | 5.51 | 4.59 | 4.17 | 91 | 3.57 | 5.82 | 4.70 | 4.63 | 99 |
| 59 | 2.47 | 4.30 | 3.39 | 4.70 | 139 | 2.44 | 4.35 | 3.40 | 4.38 | 129 |
| 60 | 4.82 | 6.66 | 5.74 | 5.33 | 93 | 4.59 | 7.42 | 6.01 | 5.74 | 96 |
| 61 | 3.86 | 5.70 | 4.78 | 4.48 | 94 | 3.65 | 6.05 | 4.85 | 4.43 | 91 |
| 62 | 2.72 | 4.56 | 3.64 | 4.70 | 129 | 2.70 | 4.65 | 3.68 | 4.52 | 123 |
| 63 | 3.00 | 4.83 | 3.91 | 3.19 | 81 | 2.91 | 4.97 | 3.94 | 3.11 | 79 |
| 64 | 4.28 | 6.11 | 5.19 | 5.26 | 101 | 4.09 | 6.63 | 5.36 | 5.45 | 102 |
| 65 | 3.17 | 5.01 | 4.09 | 4.22 | 103 | 3.05 | 5.18 | 4.11 | 4.13 | 100 |
| 66 | 2.67 | 4.51 | 3.59 | 2.92 | 81 | 2.63 | 4.57 | 3.60 | 2.92 | 81 |
| 67 | 3.47 | 5.31 | 4.39 | 4.00 | 91 | 3.30 | 5.55 | 4.43 | 4.00 | 90 |
| 68 | 3.83 | 5.67 | 4.75 | 4.96 | 104 | 3.83 | 5.67 | 4.75 | 4.96 | 104 |
| 69 | 2.90 | 4.73 | 3.82 | 2.94 | 77 | 2.89 | 4.87 | 3.88 | 2.73 | 70 |
| 70 | 2.54 | 4.38 | 3.46 | 2.26 | 65 | 2.42 | 4.43 | 3.42 | 3.80 | 111 |
| 71 | 2.33 | 4.16 | 3.24 | 2.40 | 74 | 2.46 | 4.22 | 3.34 | 2.70 | 81 |
| 72 | 3.63 | 5.46 | 4.55 | 4.73 | 104 | 3.63 | 5.79 | 4.71 | 4.92 | 105 |

| No | FEV1_  LLN | FEV1_  ULN | FEV1_  pred | FEV1_  meas | FEV1_  %pred | FEV1/FVC_LLN | FEV1/FVC_ULN | FEV1/FVC_pred | FEV1/FVC_meas | FEV1/FVC_%pred |
| --- | --- | --- | --- | --- | --- | --- | --- | --- | --- | --- |
| 1 | 1.80 | 3.37 | 2.59 | 2.68 | 104 | 60.07 | 89.27 | 74.67 | 82.80 | 111 |
| 2 | 2.46 | 3.98 | 3.22 | 1.69 | 52 | 58.26 | 79.80 | 69.03 | 73.91 | 107 |
| 3 | 1.89 | 3.52 | 2.71 | 1.54 | 57 | 60.08 | 89.00 | 74.54 | 67.97 | 91 |
| 4 | 1.81 | 3.18 | 2.50 | 1.99 | 80 | 63.94 | 90.10 | 77.02 | 80.16 | 104 |
| 5 | 2.51 | 4.26 | 3.38 | 1.96 | 58 | 64.23 | 88.51 | 76.37 | 60.50 | 79 |
| 6 | 4.08 | 2.41 | 3.25 | 1.69 | 52 | 64.69 | 88.84 | 76.77 | 51.55 | 67 |
| 7 | 2.46 | 4.01 | 3.23 | 2.36 | 73 | 66.63 | 89.33 | 77.98 | 64.83 | 83 |
| 8 | 2.31 | 3.83 | 3.07 | 2.85 | 93 | 66.02 | 89.45 | 77.74 | 61.71 | 79 |
| 9 | 2.16 | 3.66 | 2.91 | 2.57 | 89 | 65.00 | 89.50 | 77.25 | 78.59 | 102 |
| 10 | 2.38 | 4.02 | 3.20 | 2.27 | 71 | 64.69 | 88.92 | 76.80 | 76.31 | 99 |
| 11 | 1.99 | 4.76 | 3.73 | 3.70 | 99 | 61.61 | 89.04 | 75.33 | 75.33 | 100 |
| 12 | 2.04 | 3.78 | 2.91 | 2.98 | 102 | 60.08 | 88.58 | 74.33 | 76.02 | 102 |
| 13 | 1.66 | 3.02 | 2.34 | 2.19 | 93 | 62.30 | 90.16 | 76.23 | 82.74 | 109 |
| 14 | 2.53 | 4.30 | 3.42 | 3.30 | 97 | 64.11 | 88.43 | 76.27 | 80.77 | 106 |
| 15 | 2.42 | 4.36 | 3.39 | 3.56 | 105 | 61.01 | 87.86 | 74.43 | 73.32 | 99 |
| 16 | 3.01 | 4.68 | 3.85 | 3.31 | 86 | 68.64 | 89.19 | 78.91 | 69.19 | 88 |
| 17 | 2.41 | 4.31 | 3.36 | 3.03 | 90 | 61.50 | 87.99 | 74.74 | 73.04 | 98 |
| 18 | 1.83 | 3.37 | 2.60 | 2.88 | 111 | 60.97 | 89.35 | 75.16 | 77.38 | 103 |
| 19 | 2.17 | 3.89 | 3.03 | 3.27 | 108 | 61.83 | 88.61 | 75.22 | 71.18 | 95 |
| 20 | 2.24 | 3.98 | 3.11 | 3.16 | 102 | 62.07 | 88.52 | 75.30 | 77.49 | 103 |
| 21 | 2.27 | 4.08 | 3.17 | 3.94 | 124 | 61.50 | 88.30 | 74.90 | 78.08 | 104 |
| 22 | 2.40 | 4.13 | 3.27 | 1.41 | 43 | 63.70 | 88.58 | 76.14 | 56.47 | 74 |
| 23 | 2.17 | 3.89 | 3.07 | 3.21 | 105 | 60.66 | 89.67 | 74.51 | 78.33 | 105 |
| 24 | 3.15 | 5.02 | 4.09 | 2.89 | 71 | 67.03 | 88.31 | 77.67 | 67.91 | 87 |
| 25 | 2.46 | 4.08 | 3.27 | 2.86 | 87 | 65.69 | 89.03 | 77.36 | 76.77 | 99 |
| 26 | 2.71 | 4.31 | 3.51 | 3.40 | 97 | 67.67 | 89.25 | 78.46 | 78.37 | 100 |
| 27 | 2.13 | 3.77 | 2.95 | 1.83 | 62 | 62.64 | 88.91 | 75.78 | 74.19 | 98 |
| 28 | 2.95 | 4.78 | 3.87 | 2.99 | 77 | 66.25 | 88.33 | 77.29 | 65.92 | 85 |
| 29 | 2.19 | 3.75 | 2.97 | 3.00 | 101 | 64.32 | 89.24 | 76.78 | 78.02 | 102 |
| 30 | 2.08 | 3.61 | 2.85 | 2.04 | 72 | 64.03 | 89.40 | 76.72 | 80.90 | 105 |
| 31 | 1.90 | 3.38 | 2.64 | 1.39 | 52 | 62.91 | 89.59 | 76.25 | 59.96 | 79 |
| 32 | 2.34 | 4.07 | 3.21 | 3.62 | 113 | 63.21 | 88.58 | 75.89 | 73.79 | 97 |
| 33 | 1.72 | 3.19 | 2.45 | 1.84 | 75 | 60.67 | 89.66 | 75.16 | 73.98 | 98 |
| 34 | 1.86 | 3.45 | 2.66 | 1.46 | 55 | 60.35 | 89.15 | 74.75 | 59.10 | 79 |
| 35 | 2.09 | 3.65 | 2.87 | 3.03 | 106 | 63.41 | 89.23 | 76.32 | 79.80 | 105 |
| 36 | 2.44 | 4.13 | 3.28 | 2.95 | 90 | 64.60 | 88.76 | 76.68 | 76.47 | 100 |
| 37 | 1.72 | 3.16 | 2.44 | 1.52 | 62 | 61.32 | 89.78 | 75.55 | 62.81 | 83 |
| 38 | 1.94 | 3.49 | 2.71 | 1.50 | 55 | 62.03 | 89.28 | 75.65 | 54.35 | 72 |
| 39 | 2.04 | 3.66 | 2.85 | 1.54 | 54 | 62.13 | 89.01 | 75.57 | 66.25 | 88 |
| 40 | 2.33 | 3.87 | 3.10 | 2.53 | 82 | 65.88 | 89.36 | 77.62 | 76.51 | 99 |
| 41 | 2.00 | 3.64 | 2.82 | 0.67 | 24 | 61.09 | 88.91 | 75.00 | 35.02 | 47 |
| 42 | 2.24 | 3.91 | 3.08 | 2.57 | 84 | 63.29 | 88.92 | 76.06 | 74.70 | 98 |
| 43 | 1.90 | 3.50 | 2.70 | 1.18 | 43 | 60.80 | 89.10 | 74.95 | 64.91 | 87 |
| 44 | 2.27 | 3.94 | 3.10 | 2.82 | 91 | 63.52 | 88.82 | 76.17 | 77.83 | 102 |
| 45 | 2.33 | 3.85 | 3.09 | 2.12 | 69 | 66.20 | 89.46 | 77.83 | 70.69 | 91 |
| 46 | 3.52 | 5.62 | 4.57 | 4.02 | 88 | 66.33 | 87.55 | 76.94 | 66.62 | 87 |
| 47 | 2.13 | 3.83 | 2.98 | 2.93 | 98 | 61.74 | 88.70 | 75.22 | 74.98 | 100 |
| 48 | 2.27 | 4.17 | 3.22 | 3.33 | 103 | 60.11 | 88.00 | 74.05 | 74.28 | 100 |
| 49 | 2.42 | 3.91 | 3.17 | 3.34 | 106 | 67.34 | 89.64 | 78.49 | 85.37 | 109 |
| 50 | 2.82 | 4.65 | 3.74 | 3.51 | 94 | 65.34 | 88.25 | 76.79 | 74.01 | 96 |
| 51 | 2.24 | 3.85 | 3.05 | 1.46 | 48 | 64.19 | 89.07 | 76.63 | 41.33 | 54 |
| 52 | 2.58 | 4.42 | 3.50 | 3.61 | 103 | 63.62 | 88.19 | 75.91 | 73.22 | 96 |
| 53 | 2.43 | 4.41 | 3.42 | 2.94 | 86 | 60.56 | 87.73 | 74.15 | 79.47 | 107 |
| 54 | 2.07 | 3.74 | 2.90 | 1.99 | 69 | 61.44 | 88.80 | 75.12 | 75.43 | 100 |
| 55 | 2.52 | 4.09 | 3.30 | 3.47 | 105 | 66.80 | 89.27 | 78.03 | 76.29 | 98 |
| 56 | 2.41 | 4.10 | 3.25 | 3.10 | 95 | 64.34 | 88.75 | 76.55 | 76.80 | 100 |
| 57 | 1.94 | 3.52 | 2.73 | 2.06 | 75 | 61.43 | 89.14 | 75.28 | 68.59 | 91 |
| 58 | 2.75 | 4.49 | 3.62 | 3.45 | 95 | 66.03 | 88.59 | 77.31 | 74.57 | 96 |
| 59 | 1.79 | 3.29 | 2.54 | 3.03 | 119 | 61.17 | 89.52 | 75.34 | 69.34 | 92 |
| 60 | 3.49 | 5.64 | 4.56 | 4.04 | 89 | 65.53 | 87.30 | 76.42 | 70.37 | 92 |
| 61 | 2.76 | 4.62 | 3.69 | 2.60 | 70 | 64.61 | 88.14 | 76.37 | 58.55 | 77 |
| 62 | 2.02 | 3.56 | 2.79 | 3.26 | 117 | 63.16 | 89.32 | 76.24 | 72.15 | 95 |
| 63 | 2.18 | 3.80 | 2.99 | 1.77 | 59 | 63.32 | 88.99 | 76.15 | 57.10 | 75 |
| 64 | 3.14 | 5.08 | 4.11 | 4.18 | 102 | 66.00 | 87.96 | 76.98 | 76.74 | 100 |
| 65 | 2.29 | 3.96 | 3.13 | 3.15 | 101 | 63.70 | 88.82 | 76.26 | 76.35 | 100 |
| 66 | 1.95 | 3.49 | 2.72 | 2.28 | 84 | 62.48 | 89.34 | 75.91 | 78.06 | 103 |
| 67 | 2.49 | 4.24 | 3.36 | 3.10 | 92 | 64.08 | 88.51 | 76.29 | 77.32 | 101 |
| 68 | 2.78 | 4.61 | 3.70 | 4.07 | 110 | 65.04 | 88.23 | 76.64 | 82.84 | 108 |
| 69 | 2.19 | 3.76 | 2.97 | 1.77 | 59 | 64.38 | 89.24 | 76.81 | 64.71 | 84 |
| 70 | 1.74 | 3.30 | 2.52 | 2.67 | 106 | 59.40 | 89.35 | 74.38 | 70.17 | 94 |
| 71 | 1.86 | 3.26 | 2.56 | 1.37 | 53 | 63.79 | 89.92 | 76.85 | 50.66 | 66 |
| 72 | 2.85 | 4.52 | 3.68 | 3.10 | 84 | 67.78 | 89.06 | 78.42 | 62.89 | 80 |

| No | DLCO_  LLN | DLCO_  pred | DLCO_  meas | DLCO_  %pred | DLCO/VA_LLN | DLCO/VA_pred | DLCO/VA_meas | DLCO/VA_%pred |  |  |  |  |  |
| --- | --- | --- | --- | --- | --- | --- | --- | --- | --- | --- | --- | --- | --- |
| 1 | 5.28 | 7.60 | 4.75 | 62.5 | 0.74 | 1.14 | 1.08 | 94.4 |  |  |  |  |  |
| 2 | 6.37 | 8.69 | 5.79 | 66.6 | 0.82 | 1.19 | 1.34 | 112.9 |  |  |  |  |  |
| 3 | 5.68 | 8.00 | 5.65 | 70.6 | 0.77 | 1.16 | 1.29 | 111.0 |  |  |  |  |  |
| 4 | 4.74 | 7.06 | 4.52 | 64.0 | 0.76 | 1.24 | 1.23 | 99.0 |  |  |  |  |  |
| 5 | 7.18 | 9.50 | 6.05 | 64.0 | 0.93 | 1.32 | 1.20 | 91.0 |  |  |  |  |  |
| 6 | 6.80 | 9.12 | 3.48 | 38.0 | 0.92 | 1.32 | 0.80 | 61.0 |  |  |  |  |  |
| 7 | 6.58 | 8.90 | 6.59 | 74.0 | 0.94 | 1.37 | 1.04 | 76.0 |  |  |  |  |  |
| 8 | 6.22 | 8.54 | 7.32 | 86.0 | 0.91 | 1.35 | 1.12 | 83.0 |  |  |  |  |  |
| 9 | 5.52 | 7.84 | 3.02 | 38.5 | 0.82 | 1.25 | 0.68 | 54.4 |  |  |  |  |  |
| 10 | 6.62 | 8.95 | 5.51 | 61.6 | 0.91 | 1.31 | 1.10 | 84.1 |  |  |  |  |  |
| 11 | 5.79 | 8.11 | 6.62 | 82.0 | 0.80 | 1.20 | 1.21 | 100.0 |  |  |  |  |  |
| 12 | 6.30 | 8.62 | 6.48 | 75.2 | 0.81 | 1.18 | 1.11 | 94.2 |  |  |  |  |  |
| 13 | 4.68 | 7.00 | 5.40 | 77.0 | 0.73 | 1.18 | 1.26 | 107.0 |  |  |  |  |  |
| 14 | 7.23 | 9.55 | 5.87 | 62.0 | 0.93 | 1.31 | 1.16 | 88.0 |  |  |  |  |  |
| 15 | 7.52 | 9.84 | 7.15 | 73.0 | 0.90 | 1.24 | 1.10 | 89.0 |  |  |  |  |  |
| 16 | 7.95 | 10.27 | 7.75 | 75.5 | 1.05 | 1.45 | 1.65 | 113.5 |  |  |  |  |  |
| 17 | 7.36 | 9.68 | 8.65 | 89.3 | 0.89 | 1.24 | 1.22 | 98.4 |  |  |  |  |  |
| 18 | 5.26 | 7.58 | 6.14 | 81.0 | 0.75 | 1.17 | 1.07 | 91.8 |  |  |  |  |  |
| 19 | 6.48 | 8.80 | 1.97 | 22.4 | 0.85 | 1.23 | 0.33 | 26.9 |  |  |  |  |  |
| 20 | 6.65 | 8.97 | 6.63 | 74.0 | 0.87 | 1.24 | 1.15 | 93.0 |  |  |  |  |  |
| 21 | 6.92 | 9.24 | 8.22 | 89.0 | 0.87 | 1.24 | 1.18 | 95.0 |  |  |  |  |  |
| 22 | 6.94 | 9.26 | 5.78 | 60.7 | 0.91 | 1.30 | 1.08 | 83.1 |  |  |  |  |  |
| 23 | 6.72 | 9.04 | 6.17 | 68.0 | 0.85 | 1.21 | 1.04 | 85.0 |  |  |  |  |  |
| 24 | 8.57 | 10.89 | 6.83 | 63.0 | 1.05 | 1.41 | 1.11 | 79.0 |  |  |  |  |  |
| 25 | 6.71 | 9.03 | 4.98 | 55.0 | 0.93 | 1.34 | 1.00 | 75.0 |  |  |  |  |  |
| 26 | 7.17 | 9.49 | 8.60 | 91.0 | 0.99 | 1.41 | 1.55 | 110.0 |  |  |  |  |  |
| 27 | 6.16 | 8.48 | 4.87 | 57.0 | 0.84 | 1.24 | 1.10 | 89.0 |  |  |  |  |  |
| 28 | 8.09 | 10.41 | 5.29 | 51.0 | 1.01 | 1.38 | 0.86 | 62.0 |  |  |  |  |  |
| 29 | 6.05 | 8.37 | 5.21 | 62.3 | 0.86 | 1.29 | 1.00 | 78.0 |  |  |  |  |  |
| 30 | 5.76 | 8.08 | 5.72 | 70.7 | 0.84 | 1.27 | 1.34 | 105.6 |  |  |  |  |  |
| 31 | 5.21 | 7.53 | 3.94 | 52.3 | 0.78 | 1.22 | 1.02 | 83.9 |  |  |  |  |  |
| 32 | 6.81 | 9.13 | 8.30 | 91.0 | 0.89 | 1.28 | 1.11 | 86.6 |  |  |  |  |  |
| 33 | 4.79 | 7.11 | 4.27 | 60.0 | 0.71 | 1.14 | 1.02 | 90.0 |  |  |  |  |  |
| 34 | 5.52 | 7.84 | 4.38 | 55.9 | 0.77 | 1.16 | 0.93 | 79.6 |  |  |  |  |  |
| 35 | 5.85 | 8.17 | 7.58 | 92.8 | 0.84 | 1.26 | 1.32 | 104.7 |  |  |  |  |  |
| 36 | 6.85 | 9.17 | 7.27 | 79.3 | 0.92 | 1.31 | 1.35 | 103.1 |  |  |  |  |  |
| 37 | 4.77 | 7.09 | 4.31 | 61.0 | 0.72 | 1.16 | 1.01 | 87.0 |  |  |  |  |  |
| 38 | 5.52 | 7.84 | 3.64 | 46.4 | 0.79 | 1.21 | 0.84 | 70.0 |  |  |  |  |  |
| 39 | 5.92 | 8.24 | 4.34 | 52.7 | 0.82 | 1.22 | 0.92 | 75.1 |  |  |  |  |  |
| 40 | 6.27 | 8.59 | 5.91 | 69.0 | 0.90 | 1.34 | 1.07 | 80.0 |  |  |  |  |  |
| 41 | 5.94 | 8.26 | n.a. | n.a. | 0.81 | 1,20 | n.a. | n.a. |  |  |  |  |  |
| 42 | 6.47 | 8.79 | 5.15 | 58.6 | 0.88 | 1.27 | 1.16 | 91.1 |  |  |  |  |  |
| 43 | n.a. | n.a. | n.a | n.a. | n.a. | n.a. | n.a. | n.a. |  |  |  |  |  |
| 44 | 6.47 | 8.79 | 5.34 | 61.0 | 0.88 | 1.27 | 0.91 | 71.0 |  |  |  |  |  |
| 45 | 6.22 | 8.54 | 4.87 | 57.0 | 0.91 | 1.35 | 0.97 | 72.0 |  |  |  |  |  |
| 46 | 9.62 | 11.94 | 11.03 | 92.0 | 1.07 | 1.40 | 1.33 | 95.0 |  |  |  |  |  |
| 47 | 6.36 | 8.68 | 7.43 | 86.0 | 0.85 | 1.23 | 1.23 | 100.0 |  |  |  |  |  |
| 48 | 7,15 | 9.47 | 7.88 | 83.0 | 0.86 | 1.20 | 1.19 | 99.0 |  |  |  |  |  |
| 49 | 6,38 | 8.70 | 7.65 | 88.0 | 0.94 | 1.39 | 1.38 | 99.0 |  |  |  |  |  |
| 50 | 7.89 | 10.21 | 8.67 | 85.0 | 0.98 | 1.35 | 1.43 | 105.0 |  |  |  |  |  |
| 51 | 6.27 | 8.59 | 5.01 | 58.0 | 0.88 | 1.29 | 0.87 | 67.0 |  |  |  |  |  |
| 52 | 7.49 | 9.81 | 7.19 | 73.0 | 0.94 | 1.30 | 1.01 | 78.0 |  |  |  |  |  |
| 53 | 7.68 | 10.00 | 8.00 | 80.0 | 0.90 | 1.23 | 1.30 | 105.0 |  |  |  |  |  |
| 54 | 6.12 | 8.44 | 5.73 | 68.0 | 0.82 | 1.21 | 1.09 | 91.0 |  |  |  |  |  |
| 55 | 6.75 | 9.07 | 9.27 | 102.0 | 0.95 | 1.38 | 1.57 | 114.0 |  |  |  |  |  |
| 56 | 6.85 | 9.17 | 6.76 | 74.0 | 0.92 | 1.31 | 1.18 | 90.0 |  |  |  |  |  |
| 57 | 5.61 | 7.93 | 4.91 | 62.0 | 0.79 | 1.19 | 1.14 | 95.0 |  |  |  |  |  |
| 58 | n.a. | n.a. | n.a. | n.a. | n.a. | n.a. | n.a. | n.a. |  |  |  |  |  |
| 59 | 5.10 | 7.42 | 5.01 | 68.0 | 0.75 | 1.17 | 0.79 | 67.0 |  |  |  |  |  |
| 60 | 9.71 | 12.03 | 9.54 | 79.0 | 1.06 | 1.39 | 1.31 | 94.0 |  |  |  |  |  |
| 61 | 7.87 | 10.19 | 8.15 | 80.0 | 0.97 | 1.34 | 1.23 | 92.0 |  |  |  |  |  |
| 62 | 5.67 | 7.99 | 8.96 | 112.0 | 0.82 | 1.24 | 1.37 | 110.0 |  |  |  |  |  |
| 63 | 6.18 | 8.50 | 3.56 | 42.0 | 0.86 | 1.26 | 0.66 | 52.0 |  |  |  |  |  |
| 64 | 8.71 | 11.03 | 9.15 | 83.0 | 1.03 | 1.39 | 1.21 | 87.0 |  |  |  |  |  |
| 65 | 6.54 | 8.86 | 4.82 | 54.0 | 0.89 | 1.28 | 0.84 | 65.0 |  |  |  |  |  |
| 66 | 5.54 | 7.86 | 6.75 | 86.0 | 0.80 | 1.22 | 1.50 | 123.0 |  |  |  |  |  |
| 67 | 7.11 | 9.43 | 5.59 | 59.0 | 0.92 | 1.31 | 1.01 | 77.0 |  |  |  |  |  |
| 68 | 7.82 | 10.14 | 10.41 | 103.0 | 0.98 | 1.34 | 1.32 | 98.0 |  |  |  |  |  |
| 69 | 6.05 | 8.37 | 4.67 | 56.0 | 0.86 | 1.29 | 0.95 | 74.0 |  |  |  |  |  |
| 70 | 5.15 | 7.47 | 4.93 | 66.0 | 0.72 | 1.12 | 0.87 | 77.0 |  |  |  |  |  |
| 71 | n.a. | 7.48 | 4.39 | 58.7 | n.a. | 1.30 | 0.71 | 54.7 |  |  |  |  |  |
| 72 | 7.57 | 9.89 | 5.52 | 56.0 | 1.01 | 1.42 | 0.90 | 64.0 |  |  |  |  |  |

| No | Fibrosis (score) | Honey  combing | Pleural_Plaques | Pleural_  Calcinosis | PB | SC | RA | EF | DO |
| --- | --- | --- | --- | --- | --- | --- | --- | --- | --- |
| 1 | 2 | 0 | 6 | 1 | 0 | 1 | 0 | 0 | 0 |
| 2 | 3 | 1 | 3 | 0 | 0 | 0 | 0 | 2 | 0 |
| 3 | 3 | 0 | 5 | 1 | 2 | 1 | 0 | 0 | 0 |
| 4 | 4 | 0 | 6 | 1 | 2 | 0 | 1 | 0 | 0 |
| 5 | 2 | 0 | 5 | 1 | 2 | 0 | 0 | 0 | 0 |
| 6 | 3 | 0 | 4 | 1 | 0 | 5 | 0 | 0 | 0 |
| 7 | 1 | 0 | 2 | 0 | 0 | 0 | 0 | 0 | 0 |
| 8 | 1 | 0 | 0 | 0 | 0 | 0 | 0 | 0 | 0 |
| 9 | 1 | 0 | 6 | 1 | 0 | 1 | 0 | 0 | 0 |
| 10 | 3 | 0 | 1 | 0 | 0 | 0 | 0 | 0 | 0 |
| 11 | 2 | 0 | 5 | 1 | 3 | 1 | 1 | 0 | 0 |
| 12 | 4 | 0 | 6 | 1 | 0 | 2 | 0 | 0 | 0 |
| 13 | 3 | 0 | 6 | 1 | 1 | 2 | 0 | 0 | 0 |
| 14 | 6 | 0 | 6 | 1 | 5 | 2 | 1 | 0 | 0 |
| 15 | 2 | 0 | 5 | 1 | 0 | 0 | 0 | 0 | 0 |
| 16 | 2 | 0 | 4 | 1 | 2 | 0 | 1 | 0 | 0 |
| 17 | 2 | 0 | 3 | 1 | 0 | 1 | 0 | 0 | 0 |
| 18 | 4 | 0 | 4 | 1 | 0 | 1 | 0 | 0 | 0 |
| 19 | 6 | 1 | 6 | 1 | 3 | 2 | 0 | 0 | 0 |
| 20 | 4 | 0 | 6 | 1 | 1 | 3 | 0 | 0 | 0 |
| 21 | 4 | 0 | 6 | 1 | 1 | 4 | 0 | 0 | 0 |
| 22 | 4 | 0 | 6 | 1 | 4 | 1 | 1 | 0 | 0 |
| 23 | 4 | 0 | 6 | 1 | 0 | 1 | 0 | 0 | 4 |
| 24 | 1 | 0 | 0 | 0 | 1 | 0 | 0 | 0 | 0 |
| 25 | 4 | 0 | 2 | 0 | 0 | 0 | 0 | 0 | 0 |
| 26 | 1 | 0 | 0 | 0 | 0 | 0 | 0 | 0 | 0 |
| 27 | 1 | 0 | 2 | 0 | 1 | 0 | 0 | 0 | 0 |
| 28 | 3 | 0 | 0 | 0 | 5 | 0 | 0 | 0 | 0 |
| 29 | 2 | 0 | 4 | 1 | 0 | 2 | 0 | 0 | 0 |
| 30 | 3 | 0 | 5 | 1 | 1 | 4 | 1 | 2 | 0 |
| 31 | 4 | 0 | 4 | 0 | 3 | 2 | 0 | 0 | 0 |
| 32 | 2 | 0 | 2 | 1 | 0 | 0 | 0 | 0 | 0 |
| 33 | 5 | 0 | 6 | 1 | 2 | 4 | 2 | 0 | 0 |
| 34 | 7 | 0 | 5 | 1 | 0 | 5 | 1 | 0 | 0 |
| 35 | 0 | 0 | 3 | 0 | 0 | 0 | 0 | 0 | 0 |
| 36 | 1 | 0 | 4 | 0 | 0 | 0 | 0 | 2 | 0 |
| 37 | 7 | 0 | 5 | 1 | 4 | 3 | 0 | 0 | 0 |
| 38 | 4 | 0 | 4 | 1 | 2 | 3 | 0 | 0 | 0 |
| 39 | 2 | 0 | 5 | 1 | 0 | 0 | 0 | 0 | 0 |
| 40 | 4 | 0 | 3 | 0 | 0 | 0 | 0 | 0 | 2 |
| 41 | 5 | 0 | 4 | 1 | 2 | 1 | 0 | 0 | 0 |
| 42 | 4 | 0 | 6 | 1 | 1 | 2 | 0 | 0 | 0 |
| 43 | 3 | 0 | 6 | 1 | 0 | 1 | 0 | 0 | 0 |
| 44 | 4 | 0 | 6 | 1 | 0 | 3 | 0 | 0 | 0 |
| 45 | 2 | 0 | 0 | 0 | 0 | 0 | 0 | 0 | 0 |
| 46 | 1 | 0 | 2 | 0 | 0 | 0 | 0 | 0 | 0 |
| 47 | 2 | 0 | 3 | 0 | 0 | 1 | 0 | 3 | 0 |
| 48 | 1 | 0 | 6 | 1 | 0 | 1 | 0 | 0 | 0 |
| 49 | 4 | 0 | 4 | 0 | 0 | 4 | 0 | 3 | 0 |
| 50 | 0 | 0 | 1 | 0 | 0 | 0 | 0 | 0 | 0 |
| 51 | 3 | 0 | 2 | 0 | 4 | 0 | 0 | 0 | 0 |
| 52 | 2 | 0 | 0 | 0 | 0 | 0 | 0 | 0 | 0 |
| 53 | 2 | 0 | 0 | 0 | 0 | 0 | 0 | 0 | 0 |
| 54 | 10 | 0 | 6 | 0 | 0 | 1 | 0 | 0 | 0 |
| 55 | 4 | 0 | 0 | 0 | 0 | 0 | 0 | 0 | 0 |
| 56 | 2 | 0 | 0 | 0 | 1 | 0 | 0 | 0 | 0 |
| 57 | 5 | 0 | 5 | 1 | 5 | 3 | 3 | 0 | 0 |
| 58 | 1 | 0 | 0 | 0 | 0 | 1 | 0 | 0 | 0 |
| 59 | 4 | 0 | 2 | 0 | 0 | 1 | 0 | 0 | 0 |
| 60 | 2 | 0 | 0 | 0 | 0 | 0 | 0 | 0 | 0 |
| 61 | 2 | 0 | 0 | 0 | 0 | 1 | 0 | 0 | 0 |
| 62 | 2 | 0 | 2 | 1 | 0 | 2 | 0 | 0 | 0 |
| 63 | 2 | 0 | 0 | 0 | 2 | 0 | 0 | 0 | 0 |
| 64 | 3 | 0 | 2 | 1 | 1 | 3 | 0 | 0 | 4 |
| 65 | 6 | 0 | 4 | 1 | 0 | 0 | 0 | 0 | 0 |
| 66 | 1 | 0 | 5 | 1 | 0 | 0 | 0 | 0 | 0 |
| 67 | 3 | 0 | 3 | 1 | 0 | 0 | 0 | 0 | 0 |
| 68 | 5 | 0 | 1 | 0 | 3 | 1 | 0 | 0 | 0 |
| 69 | 3 | 0 | 2 | 0 | 1 | 1 | 0 | 0 | 2 |
| 70 | 1 | 0 | 1 | 0 | 0 | 0 | 0 | 0 | 0 |
| 71 | 4 | 0 | 3 | 1 | 2 | 3 | 0 | 0 | 0 |
| 72 | 1 | 0 | 0 | 0 | 0 | 0 | 0 | 0 | 0 |
