# Supplementary material for: Restrictions of VC and DLCO in relation to asbestos-related computed tomographic findings quantified by ICOERD-based parameters
Source: BMC Pulm Med. 2022 Jun 20;22:236. doi: 10.1186/s12890-022-02022-x (PMC9208103; doi:10.1186/s12890-022-02022-x)
Supplement: Supplementary file 2 — Additional file 2: Data fourfold tables of all subjects and subgroups. [file 12890_2022_2022_MOESM2_ESM.docx]

|  | **All subjects** |  |  |  |  |  |  |  |  |  |
| --- | --- | --- | --- | --- | --- | --- | --- | --- | --- | --- |
|  |  |  | **VC** | |  | **DLCO** | |  | **DLCO/VA** | |
|  |  |  | ≥LLN | <LLN |  | ≥LLN | <LLN |  | ≥LLN | <LLN |
|  |  |  |  |  |  |  |  |  |  |  |
|  |  |  |  |  |  |  |  |  |  |  |
| **Pleural plaques** | | no | 13 | 1 |  | 5 | 8 |  | 10 | 3 |
|  |  | yes | 43 | 15 |  | 19 | 37 |  | 50 | 6 |
|  | Accuracy |  | 38.89% | |  | 60.87% | |  | 23.19% | |
|  | Cohen‘s kappa | | 0.088 | |  | 0.034 | |  | -0.052 | |
|  |  |  |  |  |  |  |  |  |  |  |
|  |  |  |  |  |  |  |  |  |  |  |
| **Pulmonary fibrosis** | | no | 13 | 2 |  | 9 | 5 |  | 12 | 2 |
|  |  | yes | 43 | 14 |  | 15 | 40 |  | 48 | 7 |
|  | Accuracy |  | 37.50% | |  | 71.01% | |  | 27.54% | |
|  | Cohen‘s kappa | | 0.056 | |  | 0.292 | |  | -0.007 | |

Restrictions of VC and DLCO in relation to asbestos-related computed tomographic findings quantified by ICOERD-based parameters

Raw data: fourfold tables

| **Subgroup 1** | |  |  |  |  |  |  |  |  |  |
| --- | --- | --- | --- | --- | --- | --- | --- | --- | --- | --- |
|  |  |  | **VC** | |  | **DLCO** | |  | **DLCO/VA** | |
|  |  |  | ≥LLN | <LLN |  | ≥LLN | <LLN |  | ≥LLN | <LLN |
|  |  |  |  |  |  |  |  |  |  |  |
|  |  |  |  |  |  |  |  |  |  |  |
| **Pleural plaques** | | no | 6 | 0 |  | 1 | 5 |  | 5 | 1 |
|  |  | yes | 24 | 11 |  | 9 | 24 |  | 29 | 4 |
|  | Accuracy |  | 41.46% | |  | 64.10% | |  | 23.08% | |
|  | Cohen‘s kappa | | 0.118 | |  | -0.083 | |  | -0.016 | |
|  |  |  |  |  |  |  |  |  |  |  |
|  |  |  |  |  |  |  |  |  |  |  |
| **Pulmonary fibrosis** | | no | 5 | 1 |  | 3 | 3 |  | 5 | 1 |
|  |  | yes | 25 | 10 |  | 7 | 26 |  | 29 | 4 |
|  | Accuracy |  | 36.59% | |  | 74.36% | |  | 23.08% | |
|  | Cohen‘s kappa | | 0.045 | |  | 0.226 | |  | -0.016 | |

| **Subgroup 2** | |  |  |  |  |  |  |  |  |  |
| --- | --- | --- | --- | --- | --- | --- | --- | --- | --- | --- |
|  |  |  | **VC** | |  | **DLCO** | |  | **DLCO/VA** | |
|  |  |  | ≥LLN | <LLN |  | ≥LLN | <LLN |  | ≥LLN | <LLN |
|  |  |  |  |  |  |  |  |  |  |  |
|  |  |  |  |  |  |  |  |  |  |  |
| **Pleural plaques** | | no | 6 | 0 |  | 2 | 4 |  | 3 | 3 |
|  |  | yes | 9 | 9 |  | 2 | 15 |  | 14 | 3 |
|  | Accuracy |  | 62.50% | |  | 73.91% | |  | 26.09% | |
|  | Cohen‘s kappa | | 0.333 | |  | 0.242 | |  | -0.203 | |
|  |  |  |  |  |  |  |  |  |  |  |
|  |  |  |  |  |  |  |  |  |  |  |
| **Pulmonary fibrosis** | | no | 4 | 1 |  | 2 | 3 |  | 4 | 1 |
|  |  | yes | 11 | 8 |  | 2 | 16 |  | 13 | 5 |
|  | Accuracy |  | 50.00% | |  | 78.26% | |  | 39.13% | |
|  | Cohen‘s kappa | | 0.127 | |  | 0.311 | |  | 0.042 | |
|  | |  |  |  |  |  |  |  |  |  |
|  | **Subgroup 3** |  |  |  |  |  |  |  |  |  |
|  |  |  | **VC** | |  | **DLCO** | |  | **DLCO/VA** | |
|  |  |  | ≥LLN | <LLN |  | ≥LLN | <LLN |  | ≥LLN | <LLN |
|  |  |  |  |  |  |  |  |  |  |  |
|  |  |  |  |  |  |  |  |  |  |  |
| **Pleural plaques** | | no | 4 | 0 |  | 1 | 3 |  | 1 | 3 |
|  |  | yes | 14 | 7 |  | 3 | 17 |  | 16 | 4 |
|  | Accuracy |  | 44.00% | |  | 75.00% | |  | 20.83% | |
|  | Cohen‘s kappa | | 0.138 | |  | 0.100 | |  | -0.239 | |
|  |  |  |  |  |  |  |  |  |  |  |
|  |  |  |  |  |  |  |  |  |  |  |
| **Pulmonary fibrosis** | | no | 4 | 1 |  | 3 | 2 |  | 4 | 1 |
|  |  | yes | 14 | 6 |  | 1 | 18 |  | 13 | 6 |
|  | Accuracy |  | 4.00% | |  | 87.50% | |  | 41.67% | |
|  | Cohen‘s kappa | | 0.051 | |  | 0.591 | |  | 0.061 | |
| **Subgroup 4** | |  |  |  |  |  |  |  |  |  |
|  |  |  | **VC** | |  | **DLCO** | |  | **DLCO/VA** | |
|  |  |  | ≥LLN | <LLN |  | ≥LLN | <LLN |  | ≥LLN | <LLN |
|  |  |  |  |  |  |  |  |  |  |  |
|  |  |  |  |  |  |  |  |  |  |  |
| **Pleural plaques** | | no | 3 | 1 |  | 2 | 1 |  | 3 | 0 |
|  |  | yes | 10 | 0 |  | 8 | 2 |  | 10 | 0 |
|  | Accuracy |  | 21.43% | |  | 30.77% | |  | 23.08% | |
|  | Cohen‘s kappa | | -0.149 | |  | -0.073 | |  | 0 (not calculated) | |
|  |  |  |  |  |  |  |  |  |  |  |
|  |  |  |  |  |  |  |  |  |  |  |
| **Pulmonary fibrosis** | | no | 4 | 0 |  | 3 | 0 |  | 3 | 0 |
|  |  | yes | 9 | 1 |  | 7 | 3 |  | 10 | 0 |
|  | Accuracy |  | 35.71% | |  | 46.15% | |  | 23.08% | |
|  | Cohen‘s kappa | | 0.060 | |  | 0.165 | |  | 0 (not calculated) | |

DLCO and DLCO/VA were for three subjects not available.
